# Myocardial pathology induced by aldosterone is dependent on non-canonical activities of G protein-coupled receptor kinases

**DOI:** 10.1038/ncomms10877

**Published:** 2016-03-02

**Authors:** Alessandro Cannavo, Daniela Liccardo, Akito Eguchi, Katherine J. Elliott, Christopher J. Traynham, Jessica Ibetti, Satoru Eguchi, Dario Leosco, Nicola Ferrara, Giuseppe Rengo, Walter J. Koch

**Affiliations:** 1 Department of Pharmacology and Center for Translational Medicine, Temple University School of Medicine, N. Broad Street, Philadelphia, Pennsylvania 19140, USA; 2 Department of Physiology and Cardiovascular Research Center, Temple University School of Medicine, N. Broad Street, Philadelphia, Pennsylvania 19140, USA; 3 Department of Translational Medical Science, University of Naples Federico II, Via Pansini, 5, Naples 80131, Italy; 4 Salvatore Maugeri Foundation, IRCCS, Scientific Institute of Telese Terme,Via bagni vecchi, 1, Telese Terme, Benevento 82037, Italy

## Abstract

Hyper-aldosteronism is associated with myocardial dysfunction including induction of cardiac fibrosis and maladaptive hypertrophy. Mechanisms of these cardiotoxicities are not fully understood. Here we show that mineralocorticoid receptor (MR) activation by aldosterone leads to pathological myocardial signalling mediated by mitochondrial G protein-coupled receptor kinase 2 (GRK2) pro-death activity and GRK5 pro-hypertrophic action. Moreover, these MR-dependent GRK2 and GRK5 non-canonical activities appear to involve cross-talk with the angiotensin II type-1 receptor (AT_1_R). Most importantly, we show that ventricular dysfunction caused by chronic hyper-aldosteronism *in vivo* is completely prevented in cardiac *Grk2* knockout mice (KO) and to a lesser extent in *Grk5* KO mice. However, aldosterone-induced cardiac hypertrophy is totally prevented in *Grk5* KO mice. We also show human data consistent with MR activation status in heart failure influencing GRK2 levels. Therefore, our study uncovers GRKs as targets for ameliorating pathological cardiac effects associated with high-aldosterone levels.

## Abstract (web summary)

High aldosterone levels cause heart damage independently of its well-known effect on blood pressure. Here, Cannavo *et al*. show that aldosterone-mediated cardiac pathology involves G protein-coupled receptor (GPCR) kinase 2 (GRK2) and GRK5 that integrate signals from angiotensin II receptor (AT1R).

## Graphical abstract

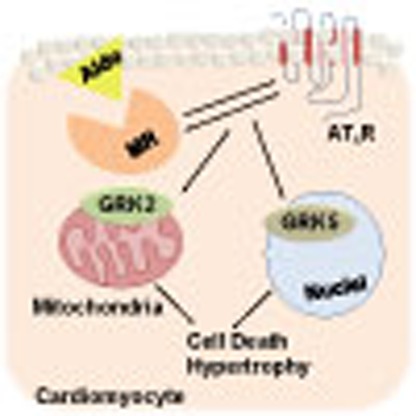

The classic neurohormonal model of heart failure (HF) is rooted in the enhancement of molecules, such as sympathetic catecholamine neurotransmitters and over-activation of the renin–angiotensin–aldosterone system (RAAS). Aldosterone, a hormone secreted by the adrenal cortex, is directly involved in the regulation of blood pressure^1^. Aldosterone has also been implicated in the pathogenesis of HF as patients have markedly elevated plasma aldosterone concentrations^2^,^3^ and increased aldosterone after myocardial infarction (MI) has been implicated in HF progression^4^. Moreover, cardiac expression of aldosterone's mineralocorticoid receptor (MR) has been shown to be elevated in HF patients^5^. In this regard, recent evidence indicates that chronic exposure to high-aldosterone levels and persistent activation of MRs can induce myocardial tissue damage via mechanisms that are independent of blood pressure elevation^6^. In fact, chronic infusion of aldosterone can lead to increased cardiac fibrosis^7^ and pathological hypertrophy^5^,^7^,^8^,^9^. Accordingly, MR antagonists such as spironolactone and eplerenone have emerged as key drugs in the armamentarium against HF to combat cardiac dysfunction associated with chronic hyper-aldosteronism^10^,^11^,^12^.

The underlying mechanisms of these deleterious effects are not completely understood and thus, there is an urgency to uncover molecular mechanisms involved in aldosterone-mediated cardiac dysfunction to identify new molecular targets and improve HF therapy. Recently, it has been shown that, through a ‘non-genomic' mechanism, aldosterone can activate NADPH oxidases (NOX2 and 4), thus increasing reactive oxygen species (ROS) and eliciting an apoptotic and fibrotic response^13^,^14^. Intriguingly, some of the effects of aldosterone in the heart can be attributed to a linkage with G protein-coupled receptor (GPCR) signalling. In particular, it appears that aldosterone can activate cross-talk between the MR^15^,^16^,^17^,^18^ and the angiotensin II (AngII) type-1 receptor (AT_1_R), a GPCR critically involved in both hypertension and HF progression^19^.

Since the AT_1_R is implicated in aldosterone-mediated cardiac dysfunction, we posited that also GPCR kinases (GRKs), as regulator of this receptor, may be involved in this deleterious mechanism. In particular, we looked at GRK2 and GRK5, the major GRKs found in the heart, since both have been linked to HF development and progression^20^,^21^, and the levels of these kinases are elevated in human failing myocardium^22^,^23^,^24^. Of note, GRK2 but not GRK5, has been shown to desensitize AngII responses in the heart^25^,^26^,^27^; however, recent evidence show that these kinases can trigger pathological myocardial signalling independent of direct GPCR regulation^27^,^28^,^29^,^30^,^31^. These non-canonical GRK activities include the unique mitochondrial localization of GRK2 promoting cell death^29^,^30^, and the translocation of GRK5 within the nucleus of myocytes promoting pathological hypertrophic gene transcription^27^,^31^. The latter indeed occurs in the heart downstream of AT_1_R activation^27^.

In this study, we have found that these non-GPCR activities of GRK2 and GRK5 are directly involved in the pathological MR-AT_1_R signalling axis in the heart. By using *in vitro* systems and *in vivo* mouse models, we have uncovered a previously unknown dependence of GRK2 and GRK5 within cardiomyocytes in aldosterone-mediated cardiac dysfunction.

## Results

### Aldosterone activates myocyte AT_1_R signalling via c-Src/β-arrestin

**Figure 1:**
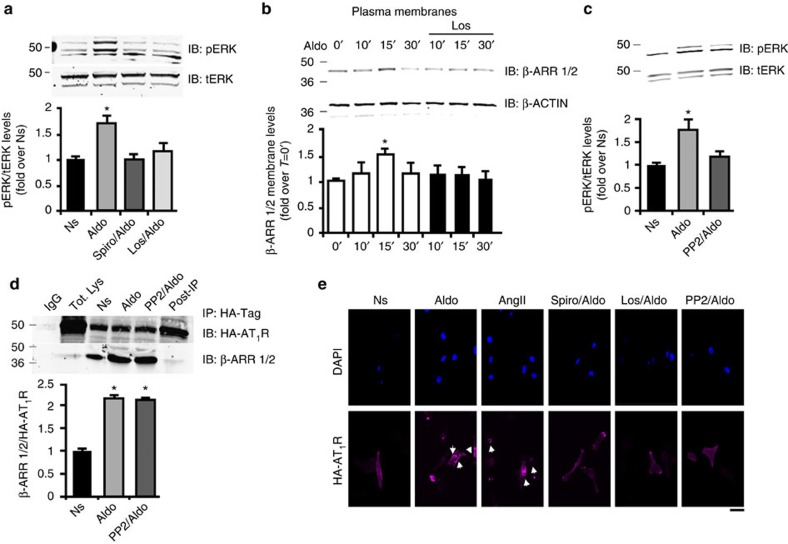
Aldosterone-mediated cross-talk between the MR and the AT_1_R. (**a**) Representative immunoblots (upper panels) and densitometric quantitative analysis (lower panel) of multiple (*n*=3) independent experiments to evaluate ERK 1/2 phosphorylation (pERK) as a ratio of activated ERK to total ERK (tERK) in neonatal rat ventricular myocytes (NRVMs) either unstimulated (Ns) or stimulated with aldosterone (Aldo 1 μM) for 15 min. Before Aldo treatment, myocytes were pre-treated with spironolactone (Spiro 10 μM) or losartan (Los 10 μM) for 30 min; *P<0.05 versus Ns. (**b**) Representative immunoblots (upper panels) and densitometric quantitative analysis (lower panel) of multiple (*n*=3) independent experiments to evaluate β-arrestin membrane recruitment in crude plasma membrane preparations from NRVMs. Shown is a time course (0–30 min) of Aldo (1 μM) treatment alone or with 30 min pre-treatment with Los (10 μM). β-ACTIN was used as loading control; *P<0.05 versus Ns. (**c**) Representative immunoblots (upper panels) and densitometric analysis (lower panel) of multiple (*n*=3) independent experiments to evaluate pERK in NRVMs Ns or stimulated with Aldo (1 μM) for 15 min. Before Aldo, a group of cells was pre-treated with PP2 (10 μM) for 30 min. tERK was used as loading control; *P<0.05 versus Ns. (**d**) Representative panels (upper panels) and densitometric analysis (lower panel) of multiple (*n*=3) independent experiments of Co-IP assay in total lysates from NRVMs infected with HA-tagged AT_1_R. Cells were Ns or stimulated with Aldo (1 μM) for 30 min. Before stimulation, a group of cells was pre-treated with the c-Src inhibitor PP2 (10 μM). Immunoprecipitated proteins (IP) for HA-tag were blotted with an antibody anti-β-arrestin 1/2 antibody; *P<0.05 versus Ns. (**e**) Representative immunofluorescence images of NRVMs infected with HA-tagged AT1R (red, lower panels). DAPI-stained myocyte nuclei are shown in the upper panel of images. Shown are cells treated for 30 min with Aldo (1 μM) or AngII (1 μM) or before Aldo, pre-treated for 30 min with Spiro (10 μM), Los (10 μM) or PP2 (10 μM). Arrows indicate receptors that are internalized. Scale bar, 20 μm. (**a**–**d**) Statistical significance between groups was determined by one-way ANOVA with Bonferroni *post hoc* correction. All data are shown as mean±s.e.m.

Aldosterone treatment of cardiomyocytes is associated with a ‘rapid' ERK 1/2 activation response that has been suggested to depend on the cross-talk between the MR and the AT_1_R^18^. Indeed, in ventricular myocytes isolated from neonatal rats (NRVMs), we found ERK activation by aldosterone peaking after 15 min (Fig. 1a). Importantly, pre-treatment of cells with spironolactone, a MR antagonist, or losartan, an AT_1_R antagonist, both could inhibit aldosterone-mediated ERK activation (Fig. 1a), indicating MR-AT_1_R cross-talk. ERK activation via GPCRs, and in particular the AT_1_R, can occur via both G protein-dependent and G protein-independent pathways^32^. The latter occurs via the combined action of, GRKs and β-ARRESTINs^33^. We therefore performed experiments to establish whether aldosterone causes AT_1_R-mediated β-arrestin recruitment and interestingly, found to be the case (Fig. 1b). Membrane β-ARRESTIN localization stimulated by aldosterone was attenuated with losartan pre-treatment, suggesting aldosterone-mediated activation of the AT_1_R endocytic machinery (Fig. 1b). Next, since C-SRC is involved in aldosterone-mediated ERK activation^34^ and is crucial for the β-ARRESTIN-mediated process of internalization and signalling transduction of the AT_1_R, even in the absence of agonist^35^, we assessed whether c-Src might play a role in this mechanism. To test this, we used the Src family kinase inhibitor, PP2 and we found that PP2-pre-treatment of cells could inhibit aldosterone-mediated ERK activation in myocytes (Fig. 1c). Further, using an adenovirus (Ad) carrying a HA-tagged AT_1_R (Ad-HA-AT_1_R) cDNA we performed a co-immunoprecipitation (Co-IP) assay to evaluate whether c-Src inhibition can affect the β-ARRESTIN recruitment to the AT_1_R. In line with previous results^35^, β-ARRESTIN recruitment was not affected in the presence of PP2 (Fig. 1d). Moreover, using an Ad-HA-AT_1_R and an Ad encoding for the MR to infect NRVMs, we found that aldosterone can induce the internalization of the AT_1_R similar to the receptor internalization induced by AngII (Fig. 1e). Interestingly, spironolactone, losartan as well as PP2 blocked aldosterone-mediated AT_1_R internalization (Fig. 1e). Thus, both AT_1_R internalization and ERK activation induced by aldosterone is C-SRC-dependent and appears to involve β-arrestins. To further support this mechanism, we used the βARKct, a peptide inhibitor of GRK2 activation via G_βγ_ sequestration^36^. βARKct expression reduced the activation of ERK by both MR and AT_1_R (Supplementary Fig. 1a).

### Critical role for GRK2 in MR-mediated myocardial pathology

**Figure 2:**
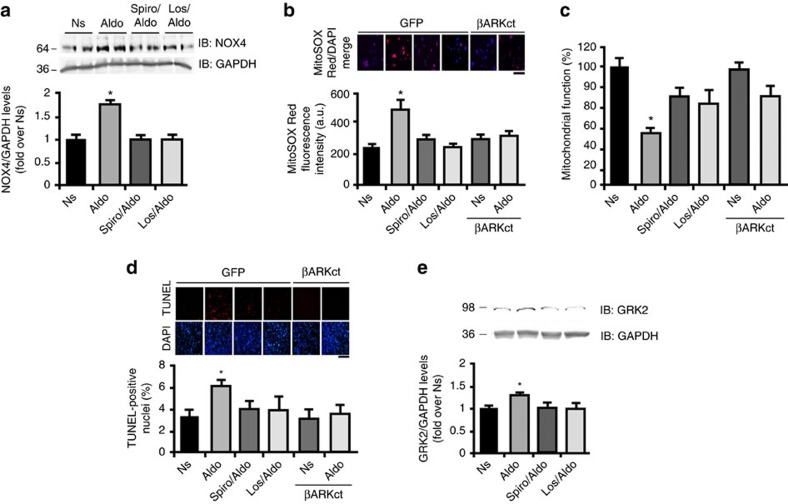
Aldosterone induced oxidative stress and apoptosis. (**a**) Representative immunoblots (upper) and densitometric quantitative analysis (lower) of panels of multiple (*n*=3) independent experiments to evaluate NOX4 protein levels in NRVMs unstimulated (Ns) or stimulated with Aldo (1 μM) for 15 min. Before Aldo, a group of cells was pre-treated with Spiro (10 μM) or Los (10 μM) for 30 min. GAPDH was used as loading control; *P<0.05 versus Ns. (**b**) Representative panels (upper) and cumulative fluorescence data (bottom) from (*n*=3) independent experiments of MitoSOX Red staining of NRVMs (∼200 cells analysed for the group for each experiment) infected with adenoviruses encoding for either GFP or βARKct. Myocytes were then Ns or stimulated for 30 min with Aldo. Before Aldo stimulation, a group of GFP cells was pre-treated (30 min) with Spiro (10 μM) or Los (10 μM); *P<0.05 versus GFP Ns. Scale bar, 50 μm. (**c**) Bar graphs showing multiple (*n*=3) independent experiment of MTT assay using NRVMs expressing βARKct or GFP after Aldo treatment for 24 h. Before Aldo stimulation, a group of GFP cells was pre-treated with Spiro (10 μM) or Los (10 μM) for 30 min; *P<0.05 versus GFP Ns. (**d**) Representative panels of TUNEL-positive myocytes (Red staining with blue DAPI staining) and quantitative data (*n*=3 independent experiments) showing NRVM apoptosis (∼1,000 cells analysed for the group for each experiment) induced by Aldo (1 μM for 24 h) with Ad-GFP infection or Ad-βARKct treatment. Before Aldo stimulation, a group of GFP cells was pre-treated with Spiro (10 μM) or Los (10 μM) for 30 min; scale bar, 200 μm. *P<0.05 versus GFP Ns. (**e**) Representative immunoblots (upper panels) and densitometric quantitative analysis (lower panel) of multiple (*n*=3) independent experiments to evaluate GRK2 protein levels in NRVMs Ns or stimulated with Aldo (1 μM) for 12 h. Before Aldo, a group of cells was pre-treated with Spiro (10 μM) or Los (10 μM) for 30 min. GAPDH was used as loading control. *P<0.05 versus Ns. (**a**–**e**) Statistical significance between groups was determined by one-way ANOVA with Bonferroni *post hoc* correction. All data are shown as mean±s.e.m.

Since the above data clearly show a myocyte MR-signalling dependence on AT_1_R and implicates GRK2 in this mechanism, we next examined whether this receptor cross-talk is involved in aldosterone-medtiated pathology in myocytes and its potential downstream signal transduction. We examined a NOX4-dependent ROS mechanism as a potential inducer of deleterious myocyte effects of aldosterone since that appears important in HF pathogenesis^13^,^14^,^37^,^38^. We found induction of NOX4 expression after aldosterone treatment of myocytes and this was blocked by both spironolactone and losartan (Fig. 2a). Aldosterone-mediated NOX4 upregulation was also blocked by βARKct expression (Supplementary Fig. 1b). Next, we assessed ROS formation in myocytes using MitoSOX Red as an indicator of NOX4 activity since it can detect superoxide production. As shown in Fig. 2b, aldosterone (30 min of exposure) increased mitochondrial ROS generation and this was blocked by antagonizing both the MR and AT_1_R as well as GRK2 via the βARKct. Further, we observed increased mitochondrial dysfunction, in response to 24 h of aldosterone using a MTT (3-(4,5-dimethylthiazol-2-yl)-2,5-diphenyltetrazolium bromide) assay and, this was blocked by MR and AT_1_R antagonism and also by GRK2 inhibition (Fig. 2c). Importantly, treatment of myocytes with aldosterone (24 h) induced a significant apoptotic response that was MR, AT_1_R and GRK2 dependent (Fig. 2d), consistent with the above signalling mechanisms.

**Figure 3:**
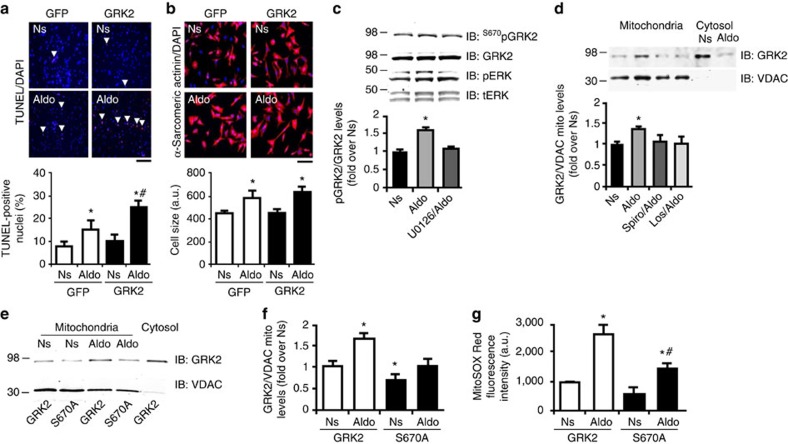
Aldosterone-mediated GRK2 mitochondrial localization in myocytes. (**a**,**b**) Representative images and quantitative data from (*n*=3) independent experiments showing (**a**) apoptotic (TUNEL staining; scale bar, 100 μm) NRVMs (∼1,000 cells analysed for the group for each experiment) or (**b**) hypertrophic (α-sarcomeric actin staining; scale bar, 50 μm) NRVMs (∼200 cells analysed for the group for each experiment) infected with either Ad-GFP or Ad-GRK2 to determine the effect of Aldo with GRK2 overexpression. Myocytes were unstimulated (Ns) or stimulated with Aldo (1 μM) for 24 (**a**) or 48 h (**b**). *P<0.05 versus GFP Ns; ^#^*P*<0.05 versus GFP Aldo. (**c**) Representative immunoblots (upper panels) and densitometric quantitative analysis (lower panel) of multiple (*n*=3) independent experiments to evaluate GRK2 phosphorylation (ser670) or ERK 1/2 phosphorylation (pERK) levels in NRVMs Ns or stimulated with Aldo (1 μM) for 15 min. Before Aldo, a group of cells was pre-treated with U0126 (3 μM) for 30 min. Total ERK (tERK) and GRK2 are shown as loading controls; *P<0.05 versus Ns. (**d**) Representative immunoblots (upper panels) and densitometric quantitative analysis (lower panel) of multiple (*n*=3) independent experiments to evaluate GRK2 levels in mitochondrial fractions purified from NRVMs that were either Ns or stimulated with Aldo (1 μM) for 30 min. Before Aldo, a group of cells was pre-treated with Spiro (10 μM) or Los (10 μM) for 30 min. VDAC was used as loading and mitochondrial purity control; *P<0.05 versus Ns. (**e**,**f**) Representative immunoblots (**e**) and denistometric quantitative analysis (**f**) of multiple (*n*=3) independent experiments to evaluate GRK2 levels in mitochondrial fractions purified from NRVMs infected with Ad-GRK2 and Ad-GRK2-S670A that were either Ns or stimulated with Aldo (1 μM) for 30 min. VDAC was used as loading and mitochondrial purity control; *P<0.05 versus GRK2 Ns. (**g**) Cumulative fluorescence data from (*n*=3) independent experiments of MitoSOX Red staining of NRVMs (∼200 cells analysed for the group for each experiment) infected with adenoviruses encoding for either GRK2 or GRK2-S670A. Myocytes were then Ns or stimulated for 30 min with Aldo. *P<0.05 versus GRK2 Ns; ^#^*P*<0.05 versus GRK2 Aldo. (**a**–**d**,**f**,**g**) Statistical significance between groups was determined by one-way ANOVA with Bonferroni *post hoc* correction. All data are shown as mean±s.e.m.

Since βARKct could block aldosterone-mediated NOX4 induction, ROS generation and myocyte apoptosis, we explored whether GRK2 played a direct role in this MR-dependent pathological signalling. Interestingly, we found that aldosterone treatment of myocytes for 12 h causes significant upregulation of GRK2 that was dependent on both MR and AT_1_R activation (Fig. 2e). To link GRK2 induction to aldosterone-mediated myocyte pathology, we overexpressed GRK2 in NRVMs and examined apoptosis, which we found to be significantly enhanced with GRK2 overexpression (Fig. 3a). Aldosterone can also induce hypertrophy of cardiomyocytes^9^ and thus, we also examined this pathological stress condition. Indeed, in control NRVMs treated with Ad encoding the green fluorescent protein (Ad-GFP), aldosterone induced increased cell size, as expected, however, unlike enhancement of apoptosis with Ad-GRK2 treatment, overexpression of GRK2 had no effect on cardiomyocyte hypertrophy in response to aldosterone (Fig. 3b).

The above data suggest that GRK2, downstream of MR activation, specifically regulates aldosterone-mediated ROS and cell death signalling. To better assess the absolute requirement of GRK2 in mitochondrial dysfunction and ROS generation, we treated cultured NRVMs with siRNA against GRK2 and as shown in Supplementary Fig. 2a,b, specific GRK2 silencing abolished the effects of aldosterone on ROS generation.

In this regard, our laboratory has shown that secondarily to ROS generation, GRK2 becomes phosphorylated by ERK at Ser670 (S670) and this causes mitochondrial localization that is dependent on the chaperone, heart shock 90 (Hsp90) protein, leading to an induction of myocyte apoptosis^29^. Accordingly, we investigated S670 phosphorylation (pS670) of GRK2 following aldosterone treatment and found that MR activation leads to GRK2 phosphorylation that is ERK dependent (Fig. 3c; Supplementary Fig. 2c). To assess whether the aldosterone-MR-ERK signalling axis could induce GRK2 translocation to mitochondria, we purified mitochondrial fractions from NRVMs treated with aldosterone and found significant mitochondrial GRK2 accumulation (Fig. 3d). Interestingly, blocking MR activation or AT_1_R activation inhibited mitochondrial translocation of GRK2 (Fig. 3d). To establish the role of GRK2 phosphorylation on ROS generation, we analysed the effects of elevated wild-type (WT) GRK2 in NRVMs versus a mutant GRK2 at Ser670 (GRK2-S670A) that cannot translocate to the mitochondria (Fig. 3e,f). As expected, following aldosterone treatment, WT GRK2 accumulated in mitochondria (Fig. 3e,f) and this was accompanied to an augmented ROS generation (Fig. 3g). Importantly, when GRK2 cannot be phosphorylated at S670, aldosterone did not lead to mitochondrial translocation of GRK2 and subsequent ROS generation was suppressed (Fig. 3g).

Previously, the ROS-, ERK- and Hsp90-dependent mitochondrial targeting of GRK2 was found to be inhibited by βARKct, since this peptide also contains S670 and ERK phosphorylation of βARKct, which competes with endogenous GRK2 for Hsp90 binding^29^. We found that aldosterone-mediated GRK2 mitochondrial translocation also appears consistent with this model as βARKct expression blocked aldosterone-mediated mitochondrial GRK2 accumulation (Supplementary Fig. 2d). As expected, βARKct expression did not inhibit mitochondrial translocation of Hsp90 downstream of aldosterone (Supplementary Fig. 2d). Further, we found that this intracellular targeting of GRK2 is dependent on the actions of AT_1_R transactivation-mediated β-arrestin function, since knockdown of β-arrestin 1/2 in NRVMs abolished GRK2 mitochondrial accumulation after aldosterone stimulation (Supplementary Fig. 2e).

Our data above suggest that aldosterone through a MR-AT_1_R-dependent pathway, induces a rapid response that increases ERK with a consequent increase in ROS generation. However, previous reports have shown that aldosterone can directly activate the oestrogen receptor (GPER) that is responsible for the rapid ERK activation observed following aldosterone stimulation^39^. To clarify whether aldosterone effects in myocytes are due to a GPER or MR-AT_1_R pathway, we used the GPER agonist E2 and the GPER antagonist G36. As expected, E2 stimulation resulted in a significant ERK activation that was efficiently blocked by G36 (Supplementary Fig. 3a). Notably, pre-treatment of cells with G36 resulted in a significant but incomplete inhibition of aldosterone-mediated ERK phosphorylation that was much less effective at blocking aldosterone signalling compared to spironolactone and losartan suggests that GPERs can be modestly involved in acute ERK activation mediated by aldosterone (Supplementary Fig. 3b). Since a recent report has shown that genetic silencing of GPER further increased aldosterone-induced ROS production with mechanisms that may suggest GPER-dependent inhibition of deleterious AT_1_R or MR signalling^40^, we explored the effects of GPER activation/inhibition on aldosterone-dependent GRK2 phosphorylation and ROS generation. Surprisingly, we found that E2 treatment of NRVMs resulted in a consistent reduction of basal phosphorylation of GRK2, at S670, that was restored by G36 (Supplementary Fig. 3c). Most importantly, we found that GPER inhibition did not affect aldosterone-dependent GRK2 phosphorylation (Supplementary Fig. 3d). In line with these results, we found that GPER activation was not able to increase ROS generation, and its inhibition with G36 also did not affect aldosterone-dependent ROS generation (Supplementary Fig. 3e). However, treatment of cells with both E2 and aldosterone resulted in an impaired cardiotoxicity compared to aldosterone alone-treated cells (Supplementary Fig. 3e). Taken together, these data suggest that GPER activation downstream of high aldosterone are not a major player in myocyte pathology as previously suggested^39^, and it appears to be due to MR-AT1R signalling.

### GRK5 is a regulator of MR/AT_1_R-mediated cardiomyocyte hypertrophy

**Figure 4:**
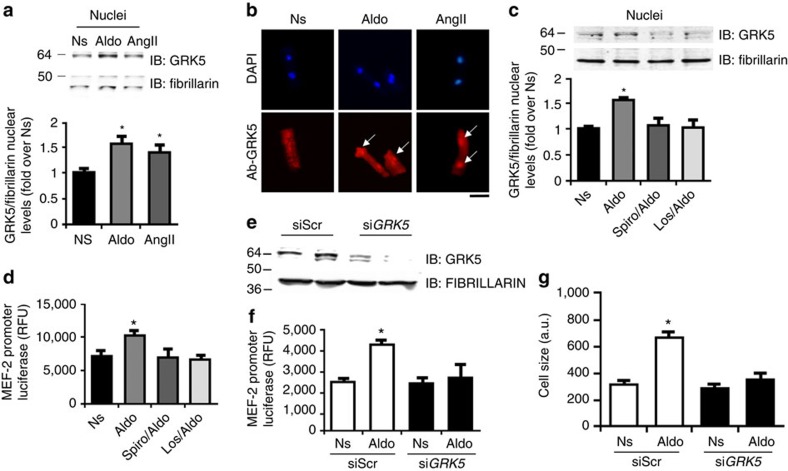
Aldosterone-mediated GRK5 nuclear localization and hypertrophic response. (**a**) Representative immunoblots (upper panels) and densitometric analysis (lower panel) of multiple independent experiments (*n*=3) to evaluate GRK5 levels in nuclear fractions purified from NRVMs, unstimulated (Ns) or stimulated with Aldo (1 μM) or AngII (1 μM) for 30 min. Fibrillarin was used as loading control; *P<0.05 versus Ns. (**b**) Representative panels of DAPI (upper) and GRK5 (bottom) immunofluorescence images in adult ventricular myocytes. The cells were Ns or stimulated with Aldo (1 μM) or AngII (1 μM) for 30 min; scale bar, 10 μm. (**c**) Representative immunoblots (upper panels) and densitometric analysis (lower panel) of multiple independent experiments (*n*=3) to evaluate GRK5 levels in nuclear fractions purified from NRVMs Ns or stimulated with Aldo (1 μM) for 30 min. Before Aldo, a group of cells was pre-treated with Spiro (10 μM) or Los (10 μM) for 30 min. Fibrillarin was used as loading control; *P<0.05 versus Ns. (**d**) Bar graph showing MEF2 reporter activity in NRVMs measured using a luciferase assay system. Cells were infected with an Ad encoding for MEF2 promoter-luciferase (Ad-MEF2-Luc) reporter construct for 48 h. Following the infection, the cells were Ns or stimulated for 24 h with Aldo (1 μM). Before Aldo, a group of cells was pre-treated with Spiro (10 μM) or Los (10 μM) for 30 min; *P<0.05 versus Ns. (**e**) Representative immunoblots showing total GRK5 levels in NRVMs transfected with siRNAs targeting GRK5 (si*GRK5*). Scrambled siRNAs (siScr) were used as control. The cells were then Ns or stimulated for 24 h with Aldo (1 μM). (**f**) Bar graph showing MEF2 reporter activity in NRVMs measured using a luciferase assay system. Cells were infected with an Ad-MEF2-Luc and transfected with siRNAs targeting GRK5 (si*GRK5*). siScr were used as control. The cells were then Ns or stimulated for 24 h with Aldo (1 μM); *P<0.05 versus siScr. (**g**) Cumulative fluorescence data from (*n*=3) independent experiments of α-sarcomeric actinin staining in NRVMs (∼200 cells analysed for the group for each experiment) transfected with siGRK5 or siScr. The cells were Ns or stimulated with Aldo (1 μM) for 48 h; *P<0.05 versus siScr Ns. (**a**,**c**,**d**,**f**,**g**) Statistical significance between groups was determined by one-way ANOVA with Bonferroni *post hoc* correction. All data are shown as mean±s.e.m.

**Figure 5:**
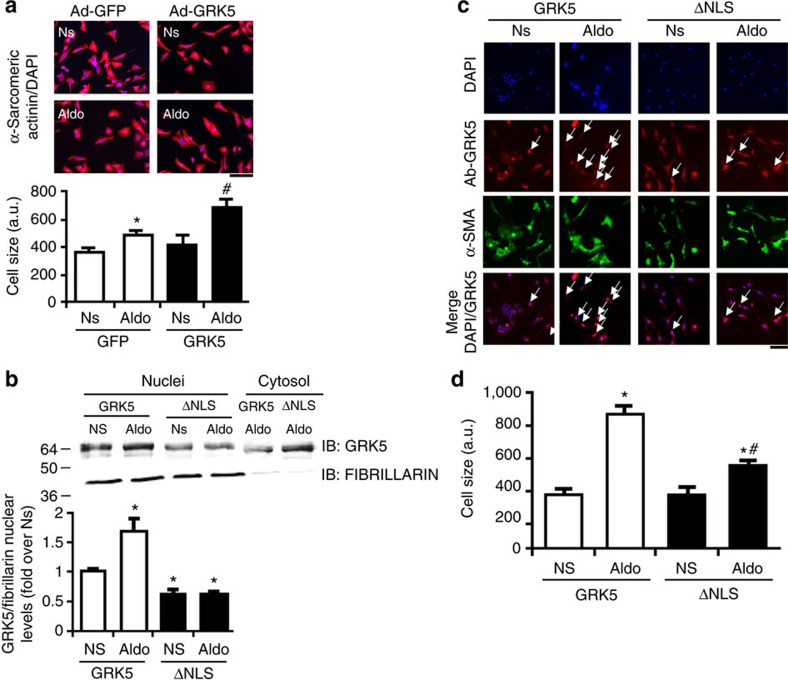
Nuclear GRK5 localization induces hypertrophic response in NRVMs. (**a**) Representative images and bar graphs showing hypertrophic response (α-sarcomeric actinin staining) in NRVMs (∼200 cells analysed for the group for each experiment) infected with Ad encoding for GFP or GRK5. The cells were Ns or stimulated with Aldo (1 μM) for 48 h; *P<0.05 versus GFP Ns; ^#^*P*<0.05 versus GFP Aldo; scale bar, 50 μm. (**b**) Representative immunoblots (upper panels) and densitometric analysis (lower panel) of multiple independent experiments (*n*=3) to evaluate GRK5 levels in nuclear fractions purified from NRVMs infected with an Ad-GRK5 or an Ad-GRK5-ΔNLS. NRVMs were Ns or stimulated with Aldo (1 μM) for 30 min. FIBRILLARIN was used as loading control; *P<0.05 versus Ns. (**c**,**d**) Representative panels of DAPI (blue), GRK5 (red) and α-sarcomeric actinin staining (α-SMA) immunofluorescence images (scale bar, 50 μm) and (**d**) bar graphs showing cumulative data of multiple independent experiments (*n*=3) to evaluate hypertrophic response in NRVMs (∼200 cells analysed for the group for each experiment) infected with Ad encoding for GRK5 or GRK5-ΔNLS. The cells were Ns or stimulated with Aldo (1 μM) for 48 h; arrows in GRK5 and in merged (DAPI/GRK5) panels indicate the nuclear localization of GRK5. *P<0.05 versus GRK5 Ns; ^#^*P*<0.05 versus GRK5 Aldo. (**a**,**b**,**d**) Statistical significance between groups was determined by one-way ANOVA with Bonferroni *post hoc* correction. All data are shown as mean±s.e.m.

The above results show that, although GRK2's non-canonical actions on mitochondrial-mediated cell death appear activated by MR-AT_1_R signalling, there was no effect on cardiac hypertrophy, prompted us to investigate another GRK involved in myocardial pathology, GRK5. The role of GRK5 in maladaptive hypertrophy has been shown to also entail non-canonical activity as a class II histone deacetylase (HDAC) kinase and nuclear factor of activated T-cells (NFAT) activator/co-factor where increased nuclear accumulation of GRK5 induces pathological gene transcription^27^,^31^,^41^. In fact, AngII, which is a potent inducer of myocardial hypertrophy, can induce GRK5 translocation to the nucleus and interestingly, the AT_1_R is not a target of GRK5-mediated desensitization^27^. Importantly, it has been previously shown that high-aldosterone levels can increase, in the heart, transcription of the myocyte enhancer factor-2 (MEF2) through the activation of Ca^2+^/calmodulin kinase II (CAMKII)^13^. Further, aldosterone can activate the calcineurin-NFAT pathway, in an AT_1_R-dependent manner^42^. Notably, these mechanisms are similar to the proposed model of pathological cardiac hypertrophy induced by the AT_1_R that is dependent on GRK5 (ref. 27). To test the hypothesis that aldosterone might induce this nuclear activity of GRK5, we first looked at NRVMs and found that aldosterone was indeed an inducer of GRK5's nuclear accumulation similar to AngII (Fig. 4a). Moreover, in adult murine ventricular myocytes we found similar induction of nuclear translocation of GRK5 via immunofluorescence (Fig. 4b). GRK5's nuclear localization after aldosterone was blocked by spironolactone treatment as well as losartan (Fig. 4c). Furthermore, we analysed the effects of GRK5 nuclear localization on MEF2 activation, and as shown in Fig. 4d, we found that in the presence of aldosterone MEF2 transcriptional activity was significantly increased compared to unstimulated cells. This increase was completely abolished by pre-treatment of cells with spironolactone and losartan (Fig. 4d). Moreover, through a GRK5 knockdown assay (Fig. 4e), we confirmed the absolute requirement of GRK5 in MEF2 activation (Fig. 4f) and in cardiac hypertrophy (Fig. 4g) downstream of aldosterone. Consistent with this, we found that overexpression of GRK5 enhanced NRVM cell size after 48 h of aldosterone treatment compared to the normal hypertrophy induced by aldosterone treatment without elevated GRK5 levels (GFP-control cells; Fig. 5a).

To better define the role of GRK5 and its nuclear activity being involved in aldosterone-mediated myocyte hypertrophy, we tested outcomes of elevated WT GRK5 versus overexpression of a mutant GRK5 lacking its nuclear localization signal (GRK5-ΔNLS). As expected, we observed that aldosterone treatment of myocytes induced consistent GRK5 nuclear localization with a robust increase in cell size growth (Fig. 5b–d). However, when GRK5-ΔNLS was overexpressed, there was no nuclear GRK5 localization and significantly diminished aldosterone-dependent cardiac hypertrophy (Fig. 5b–d).

To better evaluate the specific mechanism involved in MR-AT_1_R-dependent GRK5 nuclear translocation, we explored the ability of β-arrestin recruitment to influence the nuclear translocation of GRK5. Interstingly, we found that knockdown of β-arrestin 1/2 abolished GRK5 nuclear translocation downstream of aldosterone (Supplementary Fig. 4a). Finally, we explored the ability of aldosterone to induce the activation of Ca^2+^-calmodulin (CaM) that previously has been shown to be the nodal regulator of AngII-mediated GRK5-nuclear accumulation^31^. We pre-treated NRVMs with losartan, to inhibit the AT_1_R, and we stimulated the cells with aldosterone. Following this, we performed a Co-IP assay between GRK5 and CaM and importantly, we observed that while aldosterone induced a significant increase in CaM binding to GRK5, this was blocked by losartan (Supplementary Fig. 4b).

### *In vivo* effects of aldosterone are mediated by GRK2 and GRK5

After MI in humans, plasma aldosterone concentrations are robustly increased^43^ and the augmented concentration of this hormone can negatively affect cardiac function and HF progression^13^. To determine if any of the above *in vitro* results translate *in vivo*, we administered aldosterone to mice for 4 weeks to create a state of hyper-aldosteronism. Following chronic administration of aldosterone (2 μg per day for 4 weeks) via mini-osmotic pumps implanted subcutaneously, serum aldosterone levels were similar to the endogenous upregulation of this hormone observed in the blood serum of post-MI mice at 4 weeks (Supplementary Fig. 5a), which is consistent with reports in the literature^2^,^13^,^43^,^44^ of a ∼2-fold increase after MI, including in humans.

**Figure 6:**
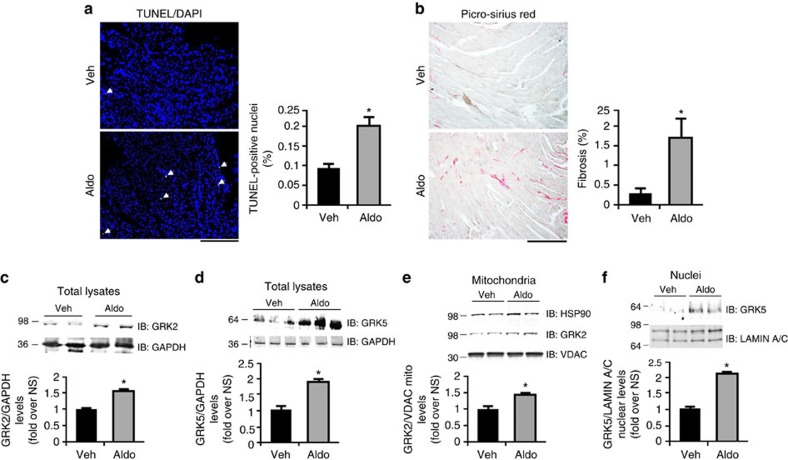
*In vivo* effects of chronic aldosterone treatment on murine myocardium. (**a**,**b**) Representative images and quantitative data from mice treated with vehicle (Veh-saline) or aldosterone (Aldo, 2 μg per day) for 4 weeks (*n*=6 mice each group). Shown in (**a**) is TUNEL/DAPI staining of cardiac sections of mice treated with Veh or Aldo and arrows indicate TUNEL-positive nuclei. Shown in (**b**) is Picro-Sirius red staining from these sections denoting cardiac fibrosis; *P<0.05 versus Veh; scale bar, 100 μm. (**c**,**d**) Representative immunoblots and quantitative data (*n*=6 mice each group) showing GRK2 (**c**) and GRK5 (**d**) protein levels in total cardiac lysates from mice treated with Aldo or Veh for 4 weeks; *P<0.05 versus Veh. (**e**) Representative immunoblots and quantitative data (*n*=6 mice each group) showing GRK2 and HSP90 levels in mitochondrial fractions purified from mouse hearts after 4 weeks of Aldo or Veh treatments. VDAC was used as mitochondrial marker and loading control; *P<0.05 versus Veh. (**f**) Representative immunoblots and quantitative data (*n*=6 mice each group) showing GRK5 levels in nuclear fractions purified from mouse hearts after 4 weeks of Aldo or Veh treatments with LAMIN A/C was used as loading control; *P<0.05 versus Veh. (**a**–**f**) Statistical significance between groups was determined by Mann-Whitney exact test. All data are shown as mean±s.e.m.

We assessed the effects of 4 weeks of aldosterone treatment on murine cardiac function. As shown in Table 1, aldosterone induced significant pathology indicated by ventricular dysfunction and adverse remodelling, including cardiac hypertrophy. At the tissue level, we found increased cardiac apoptosis, as shown by terminal deoxynucleotidyl transferase-mediated dUTP nick end labeling (TUNEL) staining (Fig. 6a), and increased myocardial fibrosis as assessed by Picro-Sirius red staining (Fig. 6b) in mice treated for 4 weeks with aldosterone. Consistent with the significant ventricular dysfunction observed in aldosterone-treated mice, GRK2 and GRK5 were upregulated in the heart (Fig. 6c,d). Interestingly, the *in vivo* increases in both GRKs, induced by aldosterone, resulted in the enhancement of their non-canonical pathological cellular locations as these hearts showed significantly more GRK2 localized to mitochondria (Fig. 6e) and significant GRK5 accumulation in nuclear fractions (Fig. 6f).

**Figure 7:**
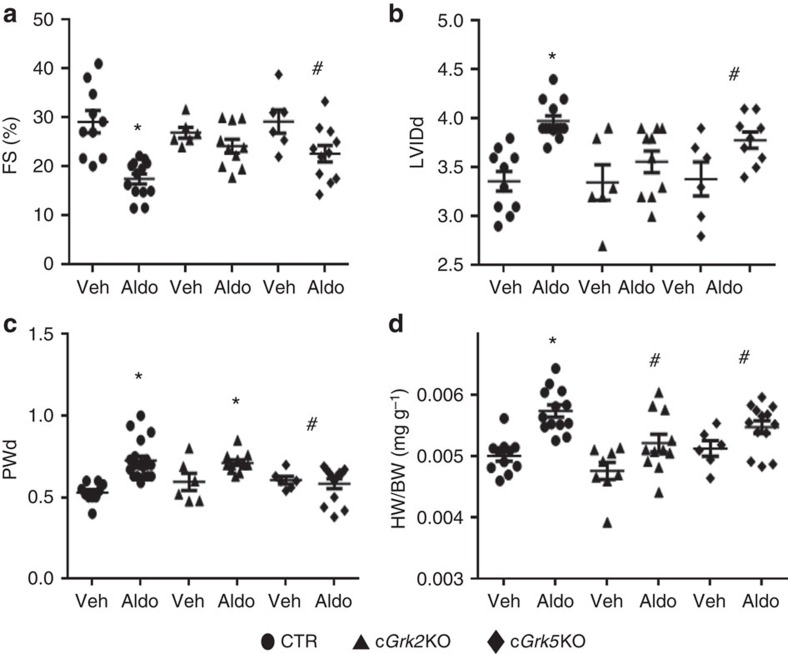
Aldosterone negatively affects *in vivo* cardiac function in a GRK2- and GRK5-dependent manner. (**a**–**c**) Dot plots showing the echocardiographic analysis of individual mice from WT (CTR), cardiac *Grk2* and *Grk5* knockout (c*Grk2* KO and c*Grk5* KO) mice after 4 weeks of Aldo (2 μg per day) or Veh (saline) treatment. Shown are measurements for (**a**) fractional shortening (FS, %); (**b**) LV internal diameter at diastole (LVIDd); and (**c**) posterior wall diastolic thickness (PWd); *P<0.05 versus CTR Veh; ^#^*P*<0.05 versus CTR Aldo. (**d**) Ratio of heart weight to body weight (HW/BW) following Aldo infusion in these mice for 4 weeks; *P<0.05 versus CTR Veh; ^#^*P*<0.05 versus CTR Aldo. (**a**–**d**) Statistical significance between groups was determined by one-way ANOVA with Bonferroni *post hoc* correction. All data are shown as mean±s.e.m. CTR, control.

To determine the absolute requirement of GRK2 and GRK5 on the pathological myocardial effects of chronic hyper-aldosteronism, we used conditional *Grk2* and *Grk5* knockout (KO) mice where these kinases were deleted specifically in cardiomyocytes^29^,^31^,^45^. Control mice (floxed alleles alone), or cardiac *Grk2* KO or *Grk5* KO mice were treated as above for 4 weeks with aldosterone and we serially assessed their cardiac function with echocardiography. We also included *αMHC-Cre* alone mice as an additional control for *Cre* expression in the heart for these experiments. Impaired cardiac function (EF) and increased left ventricular diameter (LVIDd) were similarly altered in control mice (including *αMHC-Cre* alone mice; Supplementary Fig. 5b,c) consistent with the above results after 4 weeks of hyper-aldosteronism. However, these effects were completely prevented by the loss of GRK2 in myocytes and in part attenuated by the lack of GRK5 in cardiomyocytes (Fig. 7a,b). Of interest, posterior wall (PW) thickness, a marker of ventricular growth, was increased by aldosterone in both controls and *Grk2* KO mice (Fig. 7c). However, consistent with the role of GRK5 (in the nucleus) in pathological cardiac hypertrophy, no PW increase was observed in *Grk5* KO mice (Fig. 7c). To further examine mechanisms of aldosterone-mediated cardiac dysfunction, we analysed cardiac hypertrophy via heart weight and found that indeed after 4 weeks of aldosterone infusion there was significant cardiac hypertrophy as measured by heart weight to body weight (HW/BW) ratios with control mice have the largest hearts (Fig. 7d). Of note, both *Grk2* KO and *Grk5* KO had only slight increases in HW/BW ratios after 4 weeks of aldosterone compared to controls (Fig. 7d).

**Figure 8:**
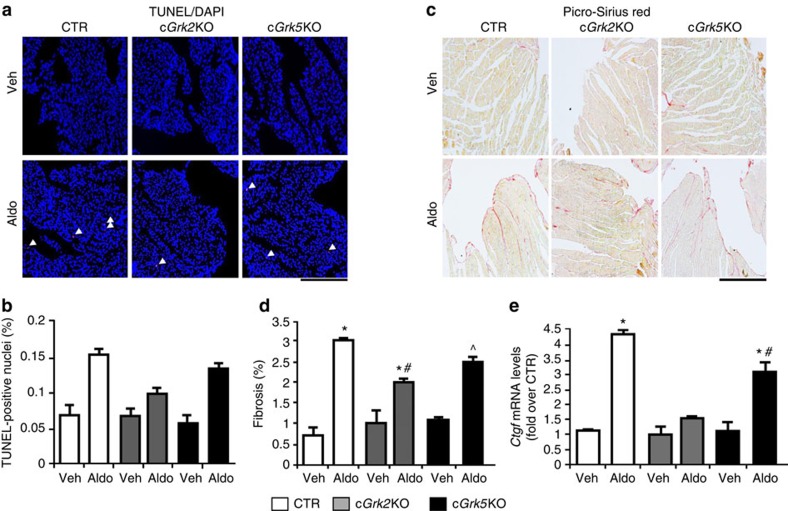
Cardiac-specific *Grk2* KO and *Grk5* KO mice are protected against the negative molecular effects induced by aldosterone (Aldo). (**a**,**b**) Representative images (upper scale bar, 200 μm) and quantitative data (*n*=8 mice each group; bottom) showing findings of myocyte cell death via TUNEL staining after 4 weeks of Aldo treatment of CTR, c*Grk2* KO and c*Grk5* KO mice; *P<0.05 versus CTR Veh; ^#^*P*<0.05 versus CTR Aldo; ^^^*P*<0.05 versus all. (**c**,**d**) Representative images (upper scale bar, 200 μm) and quantitative data (*n*=8 mice each group; bottom) showing percentage of cardiac fibrosis via Picro-Sirius red staining following 4 weeks of Aldo treatment of CTR, c*Grk2* KO and c*Grk5* KO mice; *P<0.05 versus CTR Veh; ^#^*P*<0.05 versus CTR Aldo; ^^^*P*<0.05 versus all. (**e**) Bar graph showing quantitative data of qPCR experiments to evaluate myocardial *Ctgf* mRNA levels from CTR, c*Grk2* KO and c*Grk5* KO hearts (*n*=8 each group) after Veh or Aldo treatment; *P<0.05 versus CTR Veh; ^#^*P*<0.05 versus CTR Aldo. (**b**,**d**,**e**) Statistical significance between groups was determined by one-way ANOVA with Bonferroni *post hoc* correction. All data are shown as mean±s.e.m.

To explain the differences observed between PW thickness with loss of expression of GRK2 or GRK5 in myocytes, we evaluated the effects of aldosterone on cardiac apoptosis and fibrosis. Consistent with the above results, chronic hyper-aldosteronism caused significant cardiac apoptosis and fibrosis in control mice (Fig. 8a–d) and the loss of GRK2 or GRK5 significantly affected this; however, the loss of GRK2 was significantly more effective at attenuating myocyte death and replacement fibrosis compared to the loss of GRK5 (Fig. 8a–d). As a further proof of this GRK-selective mechanism, we analysed the expression of connective tissue growth factor (CTGF), a surrogate marker of pro-fibrosis shown to be modulated directly by MR^46^. Indeed, in the hearts of aldosterone-treated control mice there was robust upregulation of *Ctgf* mRNA (Fig. 8e). In cardiac *Grk2* KO mice, there was no *Ctgf* induction while a significant but smaller reduction was observed when GRK5 was deleted in cardiomyocytes compared to control mice (Fig. 8e). These results are consistent with GRK2 being selectively involved in the fibrotic response induced by aldosterone via mitochondrial cell death, while GRK5 appears significantly involved in the hypertrophic mechanisms of pathology induced by this hormone, that is, translationally significant since both GRKs have been shown to be upregulated in human HF^23^,^24^,^45^,^47^.

To further explore the specific role of GRK2 and GRK5 in the aldosterone-dependent fibrotic response, we analysed the effects *in vitro*, in NRVMs, of the overexpression of GRK2 and GRK5 on *Ctgf* expression. Importantly, we found in control myocytes that aldosterone induced significant upregulation of CTGF (Supplementary Fig. 6a). Interestingly, GRK5 overexpression had no effect on aldosterone-mediated CTGF upregulation but GRK2 overexpression significantly enhanced this response (Supplementary Fig. 6a). Consistent with GRK2 being nodal in the fibrotic response of aldosterone in myocytes, we took conditional media from aldosterone-treated myocytes and found that the media from GRK2 overexpressing myocytes significantly enhanced the migration of cultured cardiac fibroblasts more than cultured media of control (Ad-GFP) and GRK5 overexpressing myocytes (Supplementary Fig. 6b).

These data strongly suggest that aldosterone-mediated activation of GRK2 increases the secretion of the stress-induced factor CTGF from cardiomyocytes that then acts as a paracrine factor that enhances the activation of cardiac fibroblasts contributing to fibrosis.

Finally, since we have observed that spironolactone treatment blocked the aldosterone-mediated upregulation of GRK2 in myocytes (Fig. 2e), we investigated whether MR-antagonist-treated human HF patients had altered GRK2 levels in peripheral lymphocytes. Importantly, lymphocyte GRK2 levels have been shown to mirror levels in failing human myocardium and negatively correlating with haemodynamic function^23^,^24^. Accordingly, we analysed, by immunoblot, lymphocyte GRK2 levels in human HF patients treated with spironolactone and compared these levels to a cohort of similarly diseased patients where the MR-antagonist was not used (Supplementary Table 1). Interestingly, patients treated with the MR-blocker had significantly lower GRK2 levels (Supplementary Fig. 7) consistent with this pathway having a pathophysiological influence on myocardial GRK2 levels and activity.

## Discussion

It is well known that a primary pathogenic driver of cardiac dysfunction and HF is hyper-activation of neurohormonal signalling, which propagates and maintains a vicious cycle of injury. This involves chronic sympathetic nervous system activation through increased catecholamines and also enhanced RAAS activation^2^. The catecholamines, norepinephrine and epinephrine, chronically stimulate β-adrenergic receptors (βARs) on cardiomyocytes while the RAAS promotes chronic AT_1_R activation via AngII and enhanced hyper-aldosteronism that leads to chronic MR activation. Adding to the importance of these systems and their hyper-activation, agents that block the chronic stimulation of these receptors are part of the current standard of care of HF patients, including MR antagonists. However, not all patients benefit from these drugs and new and innovative strategies for treating HF are desperately needed. Accordingly, a deeper understanding of the molecular mechanisms contributing to HF development and progression represents the best case scenario for finding new therapeutics. In this study, we have uncovered that GRK2 and GRK5 are critical molecules downstream of aldosterone and MR activation. In fact, they appear to be nodally involved in MR-mediated cardiac pathology as loss of GRK2 and GRK5 in myocytes significantly alleviates cardiac dysfunction due to hyper-aldosteronism.

**Figure 9:**
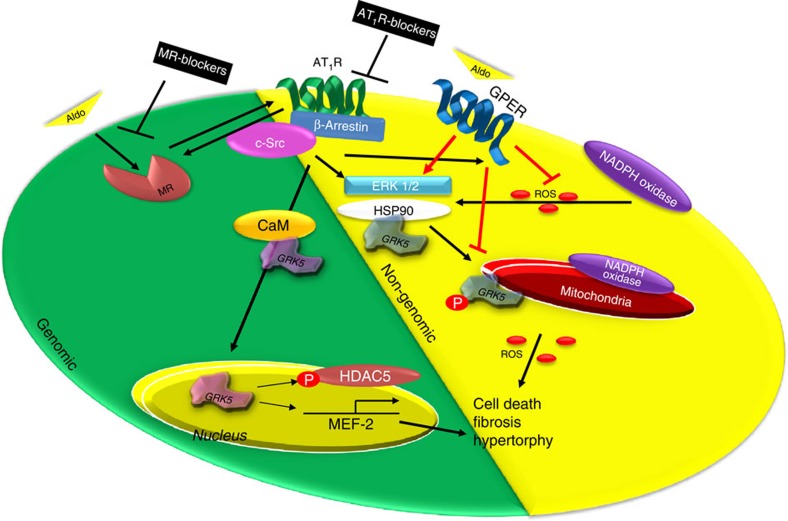
Schematic representation of aldosterone-dependent activation of GRK2 and GRK5 in NRVMs. Following binding with the MR, aldosterone recruits the β-arrestin/c-Src complex thus inducing the endocytosis of the AT_1_R and then leading to the activation of ‘genomic' and ‘non-genomic' pathways. GRK5 is part of genomic pathway since following AT_1_R activation, it binds to the Ca^2+^-CaM and translocates into the nucleus of the cardiomyocyte. Then, GRK5 acts as a HDAC5 kinase increasing the transcription of hypertrophic genes (that is, MEF2). In contrast, GRK2 is mainly involved in the non-genomic response to aldosterone stimulation. In fact, this kinase following NADPH oxidases and ERK activation is phosphorylated at ser670, and following the binding to the chaperone HSP90, GRK2 translocates to mitochondria, where it increases myocyte apoptosis. Aldosterone through GPER activation activates a parallel signalling that inhibits the phosphorylation of GRK2 and the subsequent ROS generation. Thus, GRK2 and GRK5 participate to ventricular dysfunction and heart failure progression downstream of hyper-aldosteronism. Treatment of cardiomyocytes with MR or AT_1_R antagonists or with βARKct and also knockdown of GRK2 or GRK5 them self inhibit the MR/AT_1_R signalling axis.

Our data clearly indicate that some of the pathological effects of the aldosterone are not mediated solely on MR activation, but are dependent on the transactivation of AT_1_R. We found that aldosterone treatment elicited a robust apoptotic, fibrotic and hypertrophic response in myocytes and these effects were completely abolished by selective MR- or AT_1_R-antagonism, *in vitro* (Figs 2). At the molecular level, we observed that following aldosterone-MR binding that specific GPCR-dependent endocytic machinery is recruited (Gβγ/β-arrestin/c-Src) at the plasma membrane leading to AT_1_R internalization and to the activation of ERK 1/2 and NOX4. This increase in ROS formation consequently can cause myocardial dysfunction via mitochondrial superoxide formation and a robust apoptotic response. Importantly, our data clearly show that GRK2 is vital to this response mainly through its non-canonical mitochondrial localization, which appears nodally involved in the ‘non-genomic' actions of aldosterone (Fig. 9).

In addition to GRK2 being involved in aldosterone-dependent pathology, we found that MR-AT_1_R activation leads to the nuclear translocation of GRK5, thus explaining in part the mechanism of aldosterone-mediated hypertrophic response in cardiomyocytes where this non-canonical action of GRK5 contributes to the ‘genomic' pathway of aldosterone-mediated pathology (Fig. 9).

Importantly, we also evaluated the role of the GPER that has been widely associated to the non-genomic role of aldosterone in non-myocytes and recently also in an *in vitro* model of cardiomyocytes (H9c2) (ref. 39). H9c2 cells are skeletal muscle myoblasts and although they are widely used as a surrogate of cardiomyocytes they have properties that complicate the state of MR signalling, including demonstration that H9c2 cells lose functional MRs and exhibit very low levels of aldosterone-specific MR-binding^51^. Interestingly, in H9c2 cells the GPER was found to play an important role in aldosterone-mediated deleterious signalling that could be crucial in human HF^39^.

Our data, in NRVMs show that high-aldosterone levels can activate GPER signalling but this receptor appears to have a minimal effect on myocardial pathology induced by aldosterone that instead is suggested to be fully mediated by AT_1_R-MR signalling.

Importantly, we uncovered in myocytes that aldosterone-dependent GPER activity does not explain induction of pathology, which is consistent with previous reports^39^,^52^, including in vascular smooth muscle cells where GPER stimulation can protect against hyper-activation of the MR^52^.

The most notable findings of our study were that the aldosterone-mediated negative effects on mitochondrial superoxide formation, myocyte cell death and hypertrophy were all attenuated by MR blockade with spironolactone or AT_1_R antagonism with losartan and limited aldosterone's actions by reducing the non-canonical activities of GRK2 and GRK5. Similar effects were seen using the most selective MR-antagonist eplerenone and the AT_1_R antagonist irbesartan (Supplementary Fig. 8a–c). Our data appear to be relevant to human HF as we found patients, treated with a protocol that included spironolactone, had significantly lower GRK2 levels in white blood cells, which previously has been shown to mirror cardiac levels^23^,^24^. However, although these data need more investigation, they strongly suggest that GRK2 lowering in the myocardium and lymphocytes can be associated with improved cardiac function, as previously described^24^,^48^.

Of note, previous studies have already linked GRK2 and GRK5 to HF development^20^,^21^,^27^,^31^, but, our data demonstrate for the first time that GRK2 and GRK5, acting downstream of MR activation, can induce specific myocardial pathologies. We demonstrate *in vivo* that 4 weeks of aldosterone represent a trigger stimulus for GRK2 and GRK5 upregulation and the above-discussed, non-canonical activities. These activities of GRKs appear to be significantly relevant for aldosterone-mediated HF, since cardiac *Grk2* and *Grk5* KO mice have ameliorated myocardial dysfunction. The importance of GRK-mediated cardiac pathology following chronic hyper-aldosteronism is surprising since GRKs do not regulate MR signalling. However, with the finding that MR activation transactivates AT_1_Rs in myocytes there is still a potential GPCR dependence to these pathological actions of GRK2 and GRK5. These results also demonstrate that limiting MR activation can reduce these pathological actions.

Importantly, we observed that although GRK2 clearly is a nodal determinant of aldosterone-mediated myocyte death and ultimately fibrosis *in vivo*, this kinase did not influence aldosterone-mediated cardiac hypertrophy, which is also a pathological feature of hyper-aldosteronism^8^,^9^. This is an interesting finding and demonstrates that this MR-mediated hypertrophic signalling, which also may include AT_1_R transactivation, does not involve GRK2. This is in line with previous observation since GRK2 has not been previously shown to be important in cardiac hypertrophy^49^. However, GRK5 aldosterone-MR-AT_1_R signalling is a nodal regulator of pathological cardiac growth in hyper-aldosteronism due to nuclear accumulation where its recent transcriptional influence has been revealed^27^,^41^. This was clearly shown in cardiac GRK5 KO mice where ventricular wall thickness was prevented after hyper-aldosteronism.

Recently, He *et al*.^13^ showed that aldosterone can induce direct cardiotoxic effects through the activation of the NADPH oxidases and subsequent ROS generation via actions of CAMKII acting in the nucleus after it becomes oxidized. CAMKII could become oxidized by MR activation and AT_1_R activation separately, but the co-dependence of the two receptor systems was not directly studied as in our experiments herein where GRK2 and GRK5 appear to also be nodal regulators of the pathological effects of aldosterone. Importantly, the mechanism proposed for CAMKII appears to be only responsible for cardiac rupture and mortality after MI^13^, which was not directly, evaluated in our study. The authors in this study used aldosterone infusion after MI, which raises levels to supra-physiological concentrations^13^ and in our study, levels of aldosterone were equivalent to what is seen in control mice after MI. We importantly, did not see significant deaths via rupture with physiological levels of aldosterone, but significant ventricular dysfunction and remodelling that was beneficially altered with less GRK2 and GRK5. Interestingly, although a *CamkII* KO mouse was protected from rupture^13^, it was not protected from fibrosis, which we find in *Grk2* KO mice, or by hypertrophy as seen in our *Grk5* KO mice. Therefore, GRK pathways and an oxidated-CAMKII pathway appear parallel and distinct with GRK2 being a nodal regulator of aldosterone-mediated cell death and fibrosis, and GRK5 being involved in its hypertrophic response.

Over the past two decades, GRK2 inhibition has led to prevention or reversal of several animal models of HF^20^,^21^, including most recently with βARKct gene therapy in a pre-clinical large animal model of HF^50^. Thus, GRK2 inhibition appears to be a novel therapeutic strategy for alleviating cardiac dysfunction. New data are emerging, that part of the mechanism for the beneficial effects in the heart of limiting GRK2 activity includes non-canonical roles for GRK2, including its actions on insulin signalling^28^ and mitochondrial-dependent cell death^29^,^30^. Similarly, GRK5 is also becoming an emerging novel target for its non-canonical role in the pathological hypertrophic response. Importantly, our present study demonstrates that GRK2 and GRK5 are primary determinants of cardiac dysfunction downstream of hyper-aldosteronism via their non-GPCR activities. Further, at least for GRK2, clinical treatment in human HF with spironolactone can reduce this. Accordingly, both these kinases are potential targets for HF therapy not only because of pathologic activity downstream of elevated sympathetic tone but also hyper-active RAAS signalling.

## Methods

### Agonists and inhibitors

Aldosterone was purchased from Sigma-Aldrich (A9477, St. Louis, MO, USA); AngII was purchased from Sigma-Aldrich (A9525); losartan was purchased from Sigma-Aldrich (61188); spironolactone was purchased from Sigma-Aldrich (S3378); and PP2 was purchased from Santa Cruz Biotechnology (sc-202769, Dallas, TX, USA). U0126 was purchased from Sigma-Aldrich (U120); eplerenone was purchased from Sigma-Aldrich (107724-20-9); irbesartan was purchased from Sigma-Aldrich (I2286); and PD 98059 was purchased from Sigma-Aldrich (P215). β-Estradiol was purchased from Sigma-Aldrich (E8875) and G36 was purchased from AzanoBiotech (AZ00004-G36, AzanoScientific, Albuquerque, NM, USA).

### Cell culture

Ventricular cardiomyocytes were isolated from 1- to 2-day-old neonatal rat hearts (NRVMs). Hearts were pre-washed in ADS buffer (NaCl 116 mmol l^−1^, HEPES 20 mmol l^−1^, Na_2_HPO_4_ 0.8 mmol l^−1^, glucose 5.6 mmol l^−1^, KCl 7 mmol l^−1^ and MgSO_4_-7H_2_O 0.8 mmol l^−1^, pH 7.35) to remove blood and then divided and placed in dishes with 7 ml of ADS. They were minced with sterol razor blades in small pieces and then the whole solutions were transferred in flasks and incubated at 37 °C with 7 ml enzyme solution (ADS containing pancreatin 0.6 mg ml^−1^, collagenase II 8820 U l^−1^ and CaCl_2_ 50 mmol l^−1^) for 10 min. The supernatant from this pre-digestion step was discarded and the pieces were incubated with 15 ml of digestion solution for 15-min intervals at 37 °C. After each interval, the supernatant was collected in 50 ml conical tubes containing 19 ml of F-10 media and 20% FBS pre-heated at 37 °C. The three to six collected fractions were spun down at 1,400*g* for 10 min, the supernatant was discarded and cells were washed with 5 ml of FBS for each tube. The cells were then centrifuged at 1,400*g* for 10 min and the supernatant was discarded. The resulting pellet containing the NRVMs was resuspendend in HAM's F10 complete media containing 10% horse serum (HS), 5% FBS and 1% penicilli-streptomycin (P/S), pH 7.4. The cell suspension was filtered through a 70 μm filter and pre-plated on a Nunc Nunclon 100 mm (Thermo Fisher Scientific, Waltham, MA, USA) cell culture dish for 2 h to separate the fibroblasts from the myocyte fraction. The supernatant containing mostly myocytes was collected and plated on culture dishes with Ham's F-10 complete media. The fibroblast attached to the Nunclon dishes were cultured with DMEM 1 × plus 1% P/S. Next the cells were maintained in media without HS and supplemented only with 10% FBS and P/S. Stimulation of cells with each agonist or inhibitor was performed in serum-free media. Control unstimulated cells were maintained in serum-free media.

Adult ventricular cardiac myocytes (AVMs) were isolated from LV-free wall and septum of C57/Bl6 mice. Mice were kept under anaesthesia with isoflurane and to facilitate the perfusion they were injected with heparin 100 USP into the renal artery. After removal from thoracic cavity, hearts were washed in cold perfusion buffer (NaCl 120.4 mmol l^−1^, KCl 14.7 mmol l^−1^, KH_2_PO_4_ 0.6 mmol l^−1^, Na_2_HPO_4_ 0.6 mmol l^−1^, MgSO_4_-7H_2_O 1.2 mmol l^−1^, Na-HEPES 10 mmol l^−1^, NaHCO_3_ 4.6 mmol l^−1^, taurine 30 mmol l^−1^, butanedione monoxime (BDM) 10 mmol l^−1^ and glucose 5.5 mmol l^−1^, pH 7.0). Followed cannulation on a Langendorff system, they were perfused with perfusion buffer at 37 °C for 2–3 min and then digested with digestion solution (50 ml perfusion solution with collagenase 364 U ml^−1^, 5 mg BD Difco-trypsin 250 and 20 μmol l^−1^ CaCl_2_) for 6–7 min. The atrias were removed and the ventricles were gently minced with plastic pipettes in a 100 mm dish containing 2.5 ml of digestion solution. Once the ventricles are almost completely dissolved, the digestion is stopped adding 7 ml of stopping buffer. The resulting post-digestion solutions were filtered through a 100 μm filter. The cardiomyocytes were separated from fibroblasts and smaller cells by 5-min gravity sedimentation and followed by centrifuging at 100*g* for 30 s. The supernatant was discarded and the pellet containing adult myocytes was resuspended in a stopping solution (perfusion buffer contains 250 mg BSA and 125 μmol l^−1^ CaCl_2_) and allowed to rest for 10 min. Cells were spun down with the same conditions described above and then underwent three steps to increase gradient (100 μmol l^−1^ calcium solution: 10 ml stopping buffer plus 10 μl of 100 mmol l^−1^ CaCl_2_; 400 μmol l^−1^ calcium solution: 10 ml stopping buffer plus 40 μl of 100 mmol l^−1^ CaCl_2_; and 900 μmol l^−1^ calcium solution: 10 ml stopping buffer plus 90 μl of 100 mmol l^−1^ CaCl_2_) to reach a final calcium concentration of 1.025 mmol l^−1^.

All cells were used within 2−8 h of isolation. Myocytes were plated on laminin-coated coverslips and were bathed in HEPES-buffered (20 mM, pH 7.4) medium 199 containing 1.8 mM extracellular Ca^2+^.

### Adenoviral constructs

To generate the adenoviral vector (Ad) encoding for the human MR, cDNA was first amplified from the Addgene Plasmid #23059 (ref. 53). Next, the MR PCR product was inserted into pIRES2-EGFP (Clontech, Mountain View, CA). This allows for co-expression of the GFP with the MR gene. The MRiGFP was then cloned into pAd/CMV/V5-DEST (Thermo Fisher Scientific). To generate the Ad-AT_1_R, the cDNA encoding for the rat AT_1_R with a N-terminal HA tag was subcloned into pIRESdsRed (Clontech). The AT_1_RiRED was then cloned into pAd/CMV/V5-DEST (Thermo Fisher Scientific). The Ad-vector encoding for the bovine WT *Grk2* gene (Ad-GRK2), for the WT *Grk5* gene (Ad-GRK5) and one encoding for the C-terminal region containing the last 194 amino acids of GRK2 denominated βARKct (Ad-βARKct) were previously obtained^54^,^55^. The Ad-vector encoding for the mutated form of GRK5 lacking the nuclear localization signal (GRK5-ΔNLS) was obtained previously^55^. The Ad-vector encoding the mutated form of GRK2 at Ser670 (GRK2-S670A) was previously obtained^29^. Ad-GFP was used as a control.

### Adenoviral infection

At 24 h post-isolation, NRVMs were infected with recombinant, replication-deficient adenoviruses expressing the following genes with their respective multiplicity of infection (MOI): GRK2 (50 MOI), GRK2-S670A (50 MOI), GRK5-ΔNLS (50 MOI), GRK5 (50 MOI), βARKct (50 MOI), GFP (50 MOI) HA-tagged AT_1_R (50 MOI) and MR (50 MOI). Equal particles of Ad-GFP were used to control for nonspecific adenoviral effects. Cells were cultured for 24 h before experimentation.

### GRK2, GRK5 and β-arrestin 1/2 knockdown assay

NRVMs were transfected with specific siRNAs targeting: *Grk2* (Ambion, Thermo Fisher Scientific); *Grk5* (Invitrogen-Stealth siRNA; GRK5 RSS329343, Thermo Fisher Scientific); and β-arrestin 1/2 (*Arrb1*–197273; *Arrb2*–197276; Thermo Fisher Scientific). Scrambled siRNAs were used as negative control (Thermo Fisher Scientific). The transfection was performed using 5 nmol of siRNAs together with HiPerfect Transfection Reagent (Qiagen, Germantown, Maryland, USA) according to the manufacturer's protocols.

### Western blot analysis

Cells (NRVMs and human lymphocytes) and LV samples (0.1 mg) were lysed in a RIPA buffer with protease (cOmplete-Roche, Indianapolis, IN, USA) and phosphatase inhibitors (PhosSTOP-Roche, Indianapolis, IN, USA) cocktail. Protein content was quantified with the Bio-Rad BCA protein assay (Bio-Rad Laboratories, Richmond, California, USA). Protein samples were separated by 4–20% SDS–polyacrylamide gel electrophoresis (Thermo Fisher Scientific), and proteins were transferred to nitrocellulose membrane (Bio-Rad Laboratories). After blocking, with a specific blocking buffer (Odyssey, LI-COR, Lincoln, Nebraska, USA), the membranes were incubated and probed with the first antibody at 4 °C overnight according to manufacturer's instructions. Then, the proteins were stained with a corresponding Alexa Fluor 680- (1:5,000; Thermo Fisher Scientific) or IRDye 800CW-coupled (1:5,000; Rockland Inc. Limerick, PA, USA) secondary antibody, followed by visualization of the proteins with a LI-COR infrared imager (Odyssey), and quantitative densitometric analysis was performed applying Odyssey version 1.2 infrared imaging software.

Protein levels of: GRK2 (sc-562, C-15; Santa Cruz Biotechnology, 1:1,000; 05-465; EMD Millipore, Billerica, MA, USA, 1:1,000), GRK5 (05-466; EMD Millipore, 1:2,000), GAPDH (sc-32233, 6C5; Santa Cruz Biotechnology, 1:2,000), phospho ERK 1/2 (#9106; Cell Signaling, Danvers, MA, USA, 1:1,000), total ERK 1/2 (#9102; Cell Signaling, 1:1,000), HA (sc-7392, F-7; Santa Cruz Biotechnology, 1:1,000), β-ACTIN (A5316; Sigma-Aldrich, 1:2,000), β-ARRESTIN 1/2 (#4674; Cell Signaling, 1:1,000), NOX4 (ab109225; Abcam, Cambridge, MA, USA, 1:1,000), phospho-GRK2 (pS670 44–202; Thermo Fisher Scientific, 1:1,000), voltage-dependent anion channel (VDAC) (ab14734; Abcam, 1:5,000), HSP90 (sc-13119, F-8; Santa Cruz Biotechnology, 1:1,000), LAMIN A/C (#2032; Cell Signaling, 1:1,000), FIBRILLARIN (sc-25397; Santa Cruz Biotechnology, 1:1,000) and CALMODULIN (CaM, sc-137079; Santa Cruz Biotechnology, 1:1,000) were assessed. Full scans of western blots are shown in Supplementary Fig. 9.

### Cell fractionation

Membrane proteins were isolated from LV samples (0.1 mg) and NRVMs (30 mm dish containing ∼2 × 10^6^ cells) using a homogenization buffer (0.25 M sucrose, 10 mM Tris-HCl (pH 7.4), 1 mM EDTA and protease inhibitors; cOmplete-Roche). The tissues were lysed by homogenization while the cells by scraping and pipetting. Next, both the lysates were sonicated and centrifuged for 15 min at 4 °C at 2,000 r.p.m. The supernatant was then centrifuged at 54,000 r.p.m. for 30 min at 4 °C. The pellet was washed twice using the homogenization buffer and then centrifuged again at the same speed. The washed pellet was then resuspended in PBS with 0.5% TritonX-100. The final lysate was used for western blot analysis or stored at −80 °C.

To isolate mitochondria, NRVMs (30 mm dish containing ∼2 × 10^6^ cells) and LV specimens (0.1 mg) were lysed in an ice cold mitochondrial isolation buffer (MIB) containing: 200 mmol l^−1^ mannitol, 70 mmol l^−1^ sucrose, 5 mmol l^−1^ HEPES and 1 mmol l^−1^ EGTA, pH 7.5. The MIB was supplemented with protease inhibitors (cOmplete-Roche). Cardiac specimens were first homogenized using a tissue homogenizer (PT-1200E POLYTRON), and then the lysate was subsequently homogenized on ice by passage in a 1 cc syringe with a 27 1/2 G needle (BD, Franklin lakes, NJ, USA) twenty times. NRVMs were only homogenized by passage in the syringe. Lysate was then centrifuged at 600*g* for 10 min at 4 °C. The supernatant was transferred to a new 1.5 ml tube and centrifuged again at 14,000*g* for 15 min at 4 °C. The supernatant was collected in a new tube (cytosolic fraction). While, pellet containing the mitochondria fraction was resuspended in fresh MIB buffer and centrifuged again at 14,000*g* for 15 min at 4 °C. The washed mitochondrial pellet was resuspended in MIB and directly used for protein quantification and immunoblotting. When mitochondria were taken from NRVMs, cells were washed once with ice cold Dulbecco's PBS, scraped into MIB buffer and processed as described above. Nuclear fractions were obtained from NRVMs (30 mm dish containing ∼2 × 10^6^ cells) and from LV specimens (0.1 mg). After stimulation, the cells were washed with ice cold PBS and processed using a nuclear/cytosol fractionation kit (BioVision Inc., Milpitas, CA) according to manufacturer's instructions.

### Confocal microscopy

NRVMs or AVMs washed three times in ice cold PBS and fixed in 3% paraformaldehyde (PFA) for 10 min. Then the cells were permeabilized with 0.2% Triton X-100 for 2 min. After three washes in PBS, the cells were incubated with 1% BSA for 30 min and then incubated with an anti-HA (Santa Cruz Biotechnology, 1:200) and anti-GRK5 (Santa Cruz Biotechnology, 1:200), respectively, diluted in 1% BSA. Next, the cells were incubated with the respective secondary antibodies: a mouse monoclonal to reveal the HA-tag (Alexa Fluor 647; Thermo Fisher Scientific, 1:200) and a rabbit polyclonal to reveal GRK5 (Alexa Fluor 546; Thermo Fisher Scientific 1:200). The fluorescent data sets were visualized with a Zeiss 510 confocal laser scanning microscope and analysed by LSM 510 software.

### MTT assay

MTT assay, to assess the mitochondrial function^56^, was performed in NRVMs as previously described^57^. Briefly, the cells (∼3 × 10^5^ cells) were plated in 12-well multi-well plate and were subjected to treatments (aldosterone 12 h). Following stimulation, aseptically MTT solution (5 mg ml^−1^ MTT in PBS; Sigma-Aldrich) was added in an amount equal to culture volume and the cells were incubated for 1 h. Next, MTT solvent (dimethylsulphoxide) was added to the cells in an amount equal to the original culture volume. Further, cell plates were subjected to gyratory shaker that enhanced dissolution of the MTT formazan crystals, and then the reaction was read at absorbance of 590 nm with a reference filter of 620 nm.

### MitoSOX Red staining

MitoSOX Red staining (Life Sciences, Thermo Fisher Scientific) was performed in NRVMs as previously described^58^. Briefly, after isolation ∼3 × 10^5^ cells were plated in a 30 mm dish and stimulated as described in figure legends. Then, cells were incubated for 10 min with 3 μM MitoSOX working solution. After incubation, cells were washed with warm Hank's balanced salt solution with calcium and magnesium (HBSS/Ca/Mg) and counterstained with DAPI Fluoromount-G (Southern Biotech, Birmingham, AL). Finally, cells were examined with a microscope (Nikon Eclipse Ti) and images were acquired with a digital camera (Nikon).

For each of the samples, five to six fields (NRVMs: ∼30–60 cells for field) were acquired.

### TUNEL staining

TUNEL was performed on fixed paraffin-embedded LV sections (4 μm) or on NRVMs using a commercial kit (Roche) and the assay was performed according to manufacturer's instructions. NRVMs (∼3 × 10^5^ cells) were plated in a six-well multi-well dish and stimulated as described in figure legends. TUNEL staining was visualized by specific red (Texas Red) or green fluorescence (fluorescein) and nuclei by 40,6-diamidino-2-phenylindole (DAPI; nuclear counterstain). All sections and cells were examined with a microscope (Nikon Eclipse Ni) and images were acquired with a digital camera (Nikon). For each of the samples, five to six fields (NRVMs: ∼100–300 cells for field; cardiac section: ∼600–1,000 cells for field) were acquired.

### Cell hypertrophy

After isolation, NRVMs (∼3 × 10^5^ cells) were plated in a six-well multi-well dish and stimulated as described in figure legends. NRVMs were then fixed in 3% PFA for 10 min and then washed three times in ice cold PBS and permeabilized with 0.2% Triton X-100. Then the cells were incubated with 1% BSA for 30 min and then incubated overnight at 4 °C with an anti-α-sarcomeric actinin (α-SMA, A7811, Sigma-Aldrich; 1:200) dissolved in 1% BSA. Next, cells were incubated with the respective secondary antibody (Texas Red conjugated; Sigma-Aldrich; 1:200). Cells were examined with a microscope (Nikon Eclipse Ni) and images were acquired with a digital camera (Nikon). For each of the samples, five to six fields (NRVMs: ∼50 cells for field) were acquired.

### MEF2 luciferase assay

Luciferase activity was measured using a luciferase assay system kit (Promega, Madison, WI, USA) according to manufacturer's protocol. NRVMs (24-well multi-well dish containing ∼2 × 10^5^ cells) were co-infected with the Ad-MEF2-Luc. After stimulation, cells were washed three times with ice cold PBS and collected in passive lysis buffer (Promega). Then, luciferase activity was measured using a plate reader (Infinite M1000 PRO-TECAN).

### Cell migration

Cell migration was assessed by wound-healing scratch assay as previously described^59^. After isolation, cardiac fibroblasts (∼3 × 10^5^) were plated in 12-well tissue culture plates. Twenty-four hours after plating, scratches were made using 100 μl pipette tips and the wells were washed twice with PBS. Then the cells were stimulated with conditioned media from NRVMs stimulated with aldosterone (1 μM) for 24 h. Following 3 and 12 h of stimulation, the cells were fixed in 3.7% paraformaldehyde and stained with 0.1% crystal violet staining solution. Photographs were taken on Nikon TE inverted microscope connected to a Nikon camera. Quantification of cell migration was performed by measuring the distance between 10 random points within the wound edge. Gap distance of the wound was measured using ImageJ software, and the data were normalized to the average of the wound of control cells fixed at the time of scratches.

### Animal models

All animal procedures were performed in accordance with the guidelines of the Institutional Animal Care and Use Committee of Temple University School of Medicine. For *in vivo* experiments, we used WT C57BL/6 mice and conditional mice bearing floxed *Grk2* (*Grk2-fl/fl*), which have previously been described^29^,^60^. In addition *αMHC-Cre* mice^15^ were bred on to the *Grk2-fl/fl* background to generate cardiac *Grk2* KO mice or to *Grk5-fl/fl* background to generate cardiac *Grk5* KO mice^31^, initiated by the activation of the *αMHC*-promoter and were included in the study^61^. All animals (female and males, 9–10 weeks) were bred and maintained on a C57Bl/6 background.

### Aldosterone infusion in mice

As previously described^44^, aldosterone (2 μg per mouse per day) dissolved in PBS and 5% ethanol was continuously infused subcutaneously into mice via an osmotic minipump (ALZET, DURECT Co., Cupertino, USA) for 4 weeks. A control group was infused only with PBS and 5% ethanol (vehicle). Mice were anesthetized with isoflurane (2.5% (vol/vol)) and pumps were implanted subcutaneously through a sub-scapular incision, which was then closed using 4.0 silk suture (Ethicon). After 4 weeks of infusion, transthoracic echocardiographic studies were performed, and then blood samples were collected by puncturing the heart, and heart samples were excised for pathological examination and immunohistochemistry.

### Echocardiography

Four weeks after aldosterone treatment, transthoracic echocardiography was used to assess cardiac structure and function and performed using a VisualSonics VeVo 2100 system (VisualSonics, Toronto, Ontario, Canada). Mice were anesthetized in a specific isoflurane sedation box (induction 3.0% and maintenance 1–3%). Mice were next shaved to remove hair from the ventral thorax (from the neckline to mid-chest level). Then, mice were placed in a supine position on a heated table with embedded ECG leads. During echocardiography anaesthesia was maintained throughout the procedure with 1–3% isoflurane. LV diameters and subsequently fractional shortening were evaluated with a 18–38 MHz probe. We first performed two-dimensional imaging to obtain a view along the parasternal short axis at the level of the greatest LV dimension, that include the left ventricle and a slight portion of the right ventricle. Next, we will use M-mode echocardiography, which provides a one-dimensional view, to obtain fine measurements of cardiac dimensions (LV end-diastolic and end-systolic diameter and LV anterior and PW thickness) and contractility (ejection fraction and fractional shortening). End diastole was determined at the maximal LV diastolic dimension and end systole was taken at the peak of PW motion.

### Histology

Cardiac specimens were fixed in 4% formaldehyde embedded in paraffin. After de-paraffinization and re-hydration, 5-μm-thick sections were prepared, mounted on glass slides and stained with 1% Sirius red in picric acid (Sigma-Aldrich) to detect interstitial fibrosis. The percentage of fibrosis was quantified using a software (ImageJ). All sections were examined with a microscope (Nikon Eclipse Ni) and images were acquired with a digital camera (Nikon).

### Real-time PCR

Total RNA was isolated from NRVMs and from LV specimens with TRIzol (Thermo Fisher Scientific) according to the company's instructions. After RNA isolation, cDNA was synthesized by reverse transcription of the RNA (iScript cDNA synthesis kit, Bio-Rad Laboratories). Real-time PCR was performed in duplicate on a CFX96 real-time PCR detection system (Bio-Rad Laboratories) using the SYBR Green mix (Bio-Rad Laboratories) and specific primers for mouse *Ctgf* as follows: forward 5′-GGAAGACACATTTGGCCCAG-3′; reverse 5′-TAGGTGTCCGGATGCACTTT-3′.

The expression levels of CTGF were normalized to the rRNA 18S. Specificity of PCR products was confirmed by melting curve and gel electrophoresis.

### Enzyme-linked immunosorbent assay

Aldosterone serum levels were measured in mice using a commercial kit (KA1883; Abnova, Walnut, CA, USA) and the assay was performed according to manufacturer's instructions. The assay was performed on blood serum isolated from aldosterone-treated (4 weeks) mice. Blood (300 μl) was collected by puncturing the heart. Then, blood samples were centrifuged at 1,500 r.p.m. for 15 min at room temperature and the serum was transferred to a 1.5 ml tube. Blood serum isolated from vehicle-treated mice was used as control.

### Lymphocytes GRK2 protein levels in human HF patients

We studied 127 patients with an established diagnosis of HF enroled at Federico II University (Naples, Italy) and Salvatore Maugeri Foundation, Scientific Institute of Telese Terme (Telese Terme, BN, Italy). All patients signed consent form. The patients' inclusion criteria were as follows: diagnosis of HF due to ischaemic or non-ischaemic aetiology, LV ejection fraction (LVEF) ≤45%, stable clinical conditions for at least 1 month before inclusion and guideline-based optimal pharmacotherapy. All subjects underwent a complete clinical examination (including New York Heart Association (NYHA) functional class assessment and echocardiography) and blood draw (3 ml) for lymphocyte isolation. The blood, collected in a tube containing EDTA (Vacutainer-BD, Franklin Lakes, NJ, USA), was diluted with an equal volume of PBS. The diluted blood was layered over 3 ml of Lympholyte-H Cell Separation Media (Cedarlane, Burlington, NC, USA) and centrifuged for 20 min at 800*g*. The lymphocyte layer was transferred to a new tube using a Pasteur pipette. The transferred cells were diluted with PBS and centrifuged at 800*g* for 10 min to pellet the lymphocytes. After three washes with PBS, the cells were centrifuged as above and the supernatant was removed. The resulting lymphocyte pellet was stored at −80 °C and lysed, and was analysed by immunoblot for GRK2 levels, as described above. Demographic data including age, sex, HF medications and cardiovascular risk factors and presence of comorbidities were also collected.

### Statistics

Data are expressed as mean±s.e. Statistical significance was determined by a Student's *t*-test or Mann-Whitney exact test (when sample size was <10). For multiple comparisons, one-way analysis of variance (ANOVA) followed by Bonferroni *post hoc* correction was performed. Categorical variables were expressed as proportion and compared by use of *χ*^2^ test. All data were analysed using GraphPad Prism software version 6. Statistical significance was accepted at *P*<0.05.

## Additional information

**How to cite this article:** Cannavo, A. *et al*. Myocardial pathology induced by aldosterone is dependent on non-canonical activities of G protein-coupled receptor kinases. *Nat. Commun.* 7:10877 doi: 10.1038/ncomms10877 (2016).

## Supplementary Material

Supplementary Information Supplementary Figures 1-9 and Supplementary Table 1

## Figures and Tables

**Table 1:** Effect of aldosterone treatment on LV function evaluated by echocardiography at 4 weeks after minipump implantation in WT mice.

WT C57BL/5	*N*=6	*N*=6
4 Weeks	Vehicle	Aldosterone
EF (%)	58.1±4	42±4.1*
FS (%)	30.4±2.5	22.6±4*
LVIDd	3.5±0.14	3.94±0.18*
LVIDs	2.5±0.22	2.78±0.3
PWd	0.52±0.04	0.85±0.04*
PWs	0.76±0.05	1.1±0.06*
HR	415±17.1	425±21.6
HW/BW	5.5±0.2	6.4±0.2*

*In vivo* ejection fraction (EF) and fractional shortening (FS) percentage, LV internal diameter at diastole (LVIDd), LV internal diameter at systole (LVIDs), posterior wall diastolic thickness (PWd), PW systolic thickness (PWs) and heart rate (HR) were assessed in wild-type (WT) mice (vehicle versus aldosterone). Ratio of heart weight to body weight (HW/BW) was also measured in all groups. All data are shown as mean±seem. Statistical significance between groups was determined by Mann-Whitney exact test. *N*=6 mice for each group.

^*^P<0.05 versus vehicle.

## References

[b1] Briet M. & Schiffrin E. L. Aldosterone: effects on the kidney and cardiovascular system. Nat. Rev. Nephrol. 6, 261–273 (2010). 20234356 10.1038/nrneph.2010.30

[b2] Swedberg K., Eneroth P., Kjekshus J. & Wilhelmsen L. Hormones regulating cardiovascular function in patients with severe congestive heart failure and their relation to mortality. CONSENSUS Trial Study Group. Circulation 82, 1730–1736 (1990). 2225374 10.1161/01.cir.82.5.1730

[b3] Jorde U. P. . Elevated plasma aldosterone levels despite complete inhibition of the vascular angiotensin-converting enzyme in chronic heart failure. Circulation 106, 1055–1057 (2002). 12196328 10.1161/01.cir.0000030935.89559.04

[b4] Gutierrez-Marcos F. M. . Atrial natriuretic peptide in patients with acute myocardial infarction without functional heart failure. Eur. Heart J. 12, 503–507 (1991). 1829681 10.1093/oxfordjournals.eurheartj.a059930

[b5] Yoshida M. . Mineralocorticoid receptor is overexpressed in cardiomyocytes of patients with congestive heart failure. Congest. Heart Fail. 11, 12–16 (2005). 15722665 10.1111/j.1527-5299.2005.03722.x

[b6] Marney A. M. & Brown N. J. Aldosterone and end organ damage. Clin. Sci. 113, 267–278 (2007). 17683282 10.1042/CS20070123

[b7] Brilla C. G. & Weber K. T. Mineralocorticoid excess, dietary sodium, and myocardial fibrosis. J. Lab. Clin. Med. 120, 893–901 (1992). 1453111

[b8] Takeda Y., Yoneda T., Demura M., Usukura M. & Mabuchi H. Calcineurin inhibition attenuates mineralocorticoid-induced cardiac hypertrophy. Circulation 105, 677–679 (2002). 11839620 10.1161/hc0602.104675

[b9] Yoshida K. . Excess aldosterone under normal salt diet induces cardiac hypertrophy and infiltration via oxidative stress. Hypertens. Res. 28, 447–455 (2005). 16156509 10.1291/hypres.28.447

[b10] Pitt B. . The effect of spironolactone on morbidity and mortality in patients with severe heart failure. Randomized Aldactone Evaluation Study Investigators. N. Engl. J. Med. 341, 709–717 (1999). 10471456 10.1056/NEJM199909023411001

[b11] Pitt B. . Eplerenone Post-Acute Myocardial Infarction Heart Failure Efficacy and Survival Study Investigators. Eplerenone, a selective aldosterone blocker, in patients with left ventricular dysfunction after myocardial infarction. N. Engl. J. Med. 348, 1309–1321 (2003). 12668699 10.1056/NEJMoa030207

[b12] Martinez D. V. . Cardiac damage prevention by eplerenone: comparison with low sodium diet or potassium loading. Hypertension 39, 614–618 (2002). 11882618

[b13] He B. J. . Oxidation of CaMKII determines the cardiotoxic effects of aldosterone. Nat. Med. 17, 1610–1618 (2011). 22081025 10.1038/nm.2506 PMC3332099

[b14] Hayashi H. . Aldosterone nongenomically produces NADPH oxidase-dependent reactive oxygen species and induces myocyte apoptosis. Hypertens. Res. 31, 363–375 (2008). 18360057 10.1291/hypres.31.363

[b15] de Almeida P. W. . Functional cross-talk between aldosterone and angiotensin-(1-7) in ventricular myocytes. Hypertension 61, 425–430 (2013). 23232646 10.1161/HYPERTENSIONAHA.111.199539

[b16] Lemarié C. A. . Aldosterone-induced activation of signaling pathways requires activity of angiotensin type 1a receptors. Circ. Res. 105, 852–859 (2009). 19762686 10.1161/CIRCRESAHA.109.196576

[b17] Di Zhang A. . Cross-talk between mineralocorticoid and angiotensin II signaling for cardiac remodeling. Hypertension 52, 1060–1067 (2008). 18981328 10.1161/HYPERTENSIONAHA.108.117531

[b18] Tsai C. F., Yang S. F., Chu H. J. & Ueng K. C. Cross-talk between mineralocorticoid receptor/angiotensin II type 1 receptor and mitogen-activated protein kinase pathways underlies aldosterone-induced atrial fibrotic responses in HL-1 cardiomyocytes. Int. J. Cardiol. 169, 17–28 (2013). 24120080 10.1016/j.ijcard.2013.06.046

[b19] Akazawa H., Yabumoto C., Yano M., Kudo-Sakamoto Y. & Komuro I. ARB and cardioprotection. Cardiovasc. Drugs Ther. 27, 155–160 (2013). 22538956 10.1007/s10557-012-6392-2

[b20] Cannavo A., Liccardo D. & Koch W. J. Targeting cardiac β-adrenergic signaling via GRK2 inhibition for heart failure therapy. Front. Physiol. 4, 264 (2013). 24133451 10.3389/fphys.2013.00264 PMC3783981

[b21] Sato P. Y., Chuprun J. K., Schwartz M. & Koch W. J. The evolving impact of g protein-coupled receptor kinases in cardiac health and disease. Physiol. Rev. 95, 377–404 (2015). 25834229 10.1152/physrev.00015.2014 PMC4551214

[b22] Dzimiri N., Muiya P., Andres E. & Al-Halees Z. Differential functional expression of human myocardial G protein receptor kinases in left ventricular cardiac diseases. Eur. J. Pharmacol. 489, 167–177 (2004). 15087239 10.1016/j.ejphar.2004.03.015

[b23] Iaccarino G. . Elevated myocardial and lymphocyte GRK2 expression and activity in human heart failure. Eur. Heart J. 26, 1752–1758 (2005). 16055494 10.1093/eurheartj/ehi429

[b24] Hata J. A. . Lymphocyte levels of GRK2 (betaARK1) mirror changes in the LVAD-supported failing human heart: lower GRK2 associated with improved beta-adrenergic signaling after mechanical unloading. J. Card. Fail. 12, 360–368 (2006). 16762799 10.1016/j.cardfail.2006.02.011

[b25] Rockman H. A. . Receptor-specific in vivo desensitization by the G protein-coupled receptor kinase-5 in transgenic mice. Proc. Natl Acad. Sci. USA 93, 9954–9959 (1996). 8790438 10.1073/pnas.93.18.9954 PMC38536

[b26] Rajagopal K. . Beta-arrestin2-mediated inotropic effects of the angiotensin II type 1A receptor in isolated cardiac myocytes. Proc. Natl Acad. Sci. USA 103, 16284–16289 (2006). 17060617 10.1073/pnas.0607583103 PMC1637574

[b27] Gold J. I. . Nuclear translocation of cardiac G protein-coupled receptor kinase 5 downstream of select Gq-activating hypertrophic ligands is a calmodulin-dependent process. PLoS ONE 8, e57324 (2013). 23472081 10.1371/journal.pone.0057324 PMC3589474

[b28] Ciccarelli M. . G protein-coupled receptor kinase 2 activity impairs cardiac glucose uptake and promotes insulin resistance after myocardial ischemia. Circulation 123, 1953–1962 (2011). 21518983 10.1161/CIRCULATIONAHA.110.988642 PMC3113597

[b29] Chen M. . Prodeath signaling of G protein-coupled receptor kinase 2 in cardiac myocytes after ischemic stress occurs via extracellular signal-regulated kinase-dependent heat shock protein 90-mediated mitochondrial targeting. Circ. Res. 112, 1121–1134 (2013). 23467820 10.1161/CIRCRESAHA.112.300754 PMC3908784

[b30] Fan Q. . Myocardial ablation of G protein-coupled receptor kinase 2 (GRK2) decreases ischemia/reperfusion injury through an anti-intrinsic apoptotic pathway. PLoS ONE 8, e66234 (2013). 23805205 10.1371/journal.pone.0066234 PMC3689757

[b31] Gold J. I., Gao E., Shang X., Premont R. T. & Koch W. J. Determining the absolute requirement of G protein-coupled receptor kinase 5 for pathological cardiac hypertrophy: short communication. Circ. Res. 111, 1048–1053 (2012). 22859683 10.1161/CIRCRESAHA.112.273367 PMC3752304

[b32] Kim J., Ahn S., Rajagopal K. & Lefkowitz R. J. Independent beta-arrestin2 and Gq/protein kinase Czeta pathways for ERK stimulated by angiotensin type 1A receptors in vascular smooth muscle cells converge on transactivation of the epidermal growth factor receptor. J. Biol. Chem. 284, 11953–11962 (2009). 19254952 10.1074/jbc.M808176200 PMC2673264

[b33] Claing A., Laporte S. A., Caron M. G. & Lefkowitz R. J. Endocytosis of G protein-coupled receptors: roles of G protein-coupled receptor kinases and beta-arrestin proteins. Prog. Neurobiol. 66, 61–79 (2002). 11900882 10.1016/s0301-0082(01)00023-5

[b34] Callera G. E. . c-Src-dependent nongenomic signaling responses to aldosterone are increased in vascular myocytes from spontaneously hypertensive rats. Hypertension 46, 1032–1038 (2005). 16157790 10.1161/01.HYP.0000176588.51027.35

[b35] Fessart D., Simaan M. & Laporte S. A. c-Src regulates clathrin adapter protein 2 interaction with beta-arrestin and the angiotensin II type 1 receptor during clathrin-mediated internalization. Mol. Endocrinol. 19, 491–503 (2005). 15498833 10.1210/me.2004-0246

[b36] Koch W. J. . Cardiac function in mice overexpressing the beta-adrenergic receptor kinase or a beta ARK inhibitor. Science 268, 1350–1353 (1995). 7761854 10.1126/science.7761854

[b37] Callera G. E. . Aldosterone activates vascular p38MAP kinase and NADPH oxidase via c-Src. Hypertension 45, 773–779 (2005). 15699470 10.1161/01.HYP.0000154365.30593.d3

[b38] Kuroda J. . NADPH oxidase 4 (Nox4) is a major source of oxidative stress in the failing heart. Proc. Natl Acad. Sci. USA 107, 15565–15570 (2010). 20713697 10.1073/pnas.1002178107 PMC2932625

[b39] Ashton A. W. . Role of nongenomic signaling pathways activated by aldosterone during cardiac reperfusion injury. Mol. Endocrinol. 29, 1144–1155 (2015). 26121234 10.1210/ME.2014-1410 PMC5414705

[b40] Xue B. . Estrogen receptor-beta (ERβ) in the PVN and RVLM plays an essential protective role in aldosterone/salt-induced hypertension in female rats. Hypertension 61, 1255–1262 (2013). 23608653 10.1161/HYPERTENSIONAHA.111.00903 PMC3893074

[b41] Hullmann J. E. . GRK5-mediated exacerbation of pathological cardiac hypertrophy involves facilitation of nuclear NFAT activity. Circ. Res. 115, 976–985 (2014). 25332207 10.1161/CIRCRESAHA.116.304475 PMC4258119

[b42] Colella M. & Pozzan T. Cardiac cell hypertrophy *in vitro*: role of calcineurin/NFAT as Ca^2+^ signal integrators. Ann. NY Acad. Sci. 1123, 64–68 (2008). 18375578 10.1196/annals.1420.008

[b43] Weber K. T. Aldosterone in congestive heart failure. N. Engl. J. Med. 345, 1689–1697 (2001). 11759649 10.1056/NEJMra000050

[b44] Keidar S. . Aldosterone administration to mice stimulates macrophage NADPH oxidase and increases atherosclerosis development: a possible role for angiotensin-converting enzyme and the receptors for angiotensin II and aldosterone. Circulation 109, 2213–2220 (2004). 15123520 10.1161/01.CIR.0000127949.05756.9D

[b45] Raake P. W. . G protein-coupled receptor kinase 2 ablation in cardiac myocytes before or after myocardial infarction prevents heart failure. Circ. Res. 103, 413–422 (2008). 18635825 10.1161/CIRCRESAHA.107.168336 PMC2679955

[b46] Messaoudi S. . Aldosterone-specific activation of cardiomyocyte mineralocorticoid receptor *in vivo*. Hypertension 61, 361–367 (2013). 23297371 10.1161/HYPERTENSIONAHA.112.198986

[b47] Dzimiri N. . Differential functional expression of human myocardial G protein receptor kinases in left ventricular cardiac diseases. Eur. J. Pharmacol. 489, 167–177 (2004). 15087239 10.1016/j.ejphar.2004.03.015

[b48] Rengo G. . Reduction of lymphocyte G protein-coupled receptor kinase-2 (GRK2) after exercise training predicts survival in patients with heart failure. Eur. J. Prev. Cardiol. 21, 4–11 (2014). 23689525 10.1177/2047487313491656

[b49] Tachibana H., Naga Prasad S. V., Lefkowitz R. J., Koch W. J. & Rockman H. A. Level of beta-adrenergic receptor kinase 1 inhibition determines degree of cardiac dysfunction after chronic pressure overload-induced heart failure. Circulation 111, 591–597 (2005). 15668342 10.1161/01.CIR.0000142291.70954.DF

[b50] Raake P. W. . AAV6.βARKct cardiac gene therapy ameliorates cardiac function and normalizes the catecholaminergic axis in a clinically relevant large animal heart failure model. Eur. Heart J. 34, 1437–1447 (2013). 22261894 10.1093/eurheartj/ehr447 PMC3653122

[b51] Fejes-Tóth G. & Náray-Fejes-Tóth A. Early aldosterone-regulated genes in cardiomyocytes: clues to cardiac remodeling? Endocrinology 148, 1502–1510 (2007). 17234708 10.1210/en.2006-1438

[b52] Jaffe I. Z. & Mendelsohn M. E. Angiotensin II and aldosterone regulate gene transcription via functional mineralocortocoid receptors in human coronary artery smooth muscle cells. Circ. Res. 96, 643–650 (2005). 15718497 10.1161/01.RES.0000159937.05502.d1

[b53] Miller C. A. 3rd, Tan X., Wilson M., Bhattacharyya S. & Ludwig S. Single plasmids expressing human steroid hormone receptors and a reporter gene for use in yeast signaling assays. Plasmid 63, 73–78 (2010). 19962400 10.1016/j.plasmid.2009.11.003 PMC2819589

[b54] Lymperopoulos A., Rengo G., Zincarelli C., Soltys S. & Koch W. J. Modulation of adrenal catecholamine secretion by *in vivo* gene transfer and manipulation of G protein-coupled receptor kinase-2 activity. Mol. Ther. 16, 302–307 (2008). 18223549 10.1038/sj.mt.6300371

[b55] Martini J. S. . Uncovering G protein-coupled receptor kinase-5 as a histone deacetylase kinase in the nucleus of cardiomyocytes. Proc. Natl Acad. Sci. USA 105, 12457–12462 (2008). 18711143 10.1073/pnas.0803153105 PMC2527933

[b56] Brand M. D. & Nicholls D. G. Assessing mitochondrial dysfunction in cells. Biochem. J. 435, 297–312 (2011). 21726199 10.1042/BJ20110162 PMC3076726

[b57] Cannavo A. . Prothymosin alpha protects cardiomyocytes against ischemia-induced apoptosis via preservation of Akt activation. Apoptosis 18, 1252–1261 (2013). 23857453 10.1007/s10495-013-0876-9

[b58] Perrino C. . Genetic deletion of uncoupling protein 3 exaggerates apoptotic cell death in the ischemic heart leading to heart failure. J. Am. Heart Assoc. 2, e000086 (2013). 23688674 10.1161/JAHA.113.000086 PMC3698767

[b59] Cannavo A. . βARKct gene-therapy improves β2-adrenergic receptor-dependent neoangiogenesis following hindlimb ischemia. J. Pharmacol. Exp. Ther. 115, 228411 (2015).

[b60] Matkovich S. J. . Cardiac-specific ablation of G-protein receptor kinase 2 redefines its roles in heart development and beta-adrenergic signaling. Circ. Res. 99, 996–1003 (2006). 17008600 10.1161/01.RES.0000247932.71270.2c

[b61] Agah R. . Gene recombination in postmitotic cells. Targeted expression of Cre recombinase provokes cardiac-restricted, site-specific rearrangement in adult ventricular muscle *in vivo*. J. Clin. Invest. 100, 169–179 (1997). 9202069 10.1172/JCI119509 PMC508177

