# Supplementary material for: Myocardial pathology induced by aldosterone is dependent on non-canonical activities of G protein-coupled receptor kinases
Source: Nat Commun. 2016 Mar 2;7:10877. doi: 10.1038/ncomms10877 (PMC4778065; doi:10.1038/ncomms10877)
Supplement: Supplementary Information — Supplementary Figures 1-9 and Supplementary Table 1 [file ncomms10877-s1.pdf]

Supplementary Information

Supplementary Figure 1

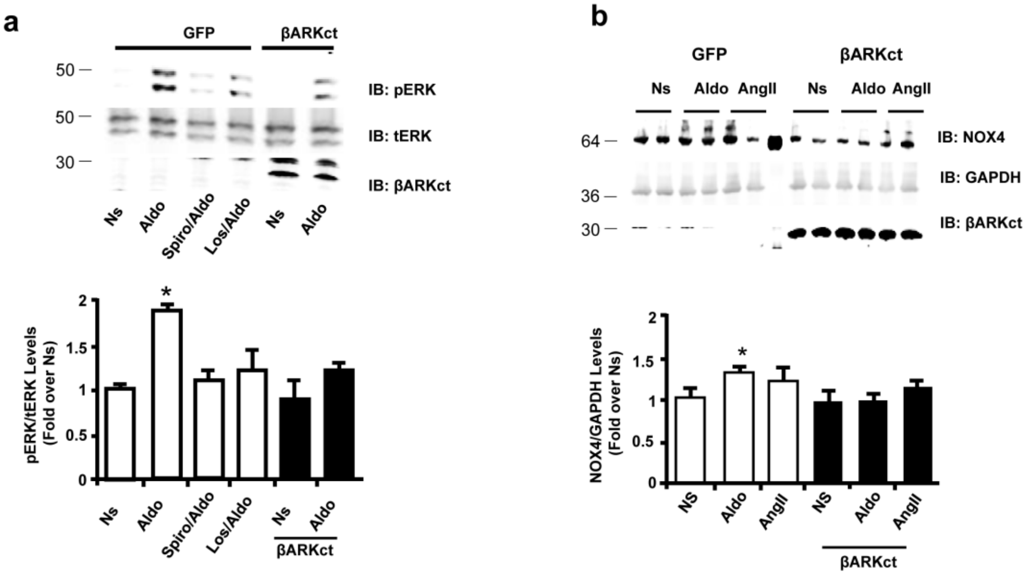

Supplementary Figure 1: βARKct abolishes the MR/AT$_1$R-dependent activation of ERK and the NOX4 up-regulation. a) Representative immunoblot (upper) and densitometric analysis (lower) of multiple (n=3) independent experiment showing ERK1/2 phosphorylation (pERK) levels following aldosterone (Aldo - 1 µM) stimulation of neonatal rat ventricular myocytes (NRVMs) for 15 min or Ns. NRVMs were infected with an Adenovirus (Ad) carrying GFP or βARKct. In some GFP samples, myocytes were pre-treated for 30 min, with spironolactone (Spiro - 10 µM) or losartan (Los - 10µmol/L) prior to Aldo addition. Total ERK (tERK) was used as loading control and pERK normalized to tERK levels. *, p<0.05 vs GFP NS. **b)** Representative immunoblot (upper) and densitometric analysis (lower) of multiple (n=3) independent experiment showing total NOX4 levels following 24 hrs of Aldo (1 µM) or Angiotensin II (Ang II - 1 µM) stimulation of NRVMs. Prior to Aldo stimulation, myocytes were infected with Ad-GFP (white bars) or Ad-βARKct (Black bars) as indicated. GAPDH was used as loading control and NOX4 normalized. *, p<0.05 vs GFP NS. **a-b**) Statistical significance between groups was determined by one-way ANOVA with Bonferroni post-hoc correction. All data are shown as the means±s.e.m.

Supplementary Figure 2

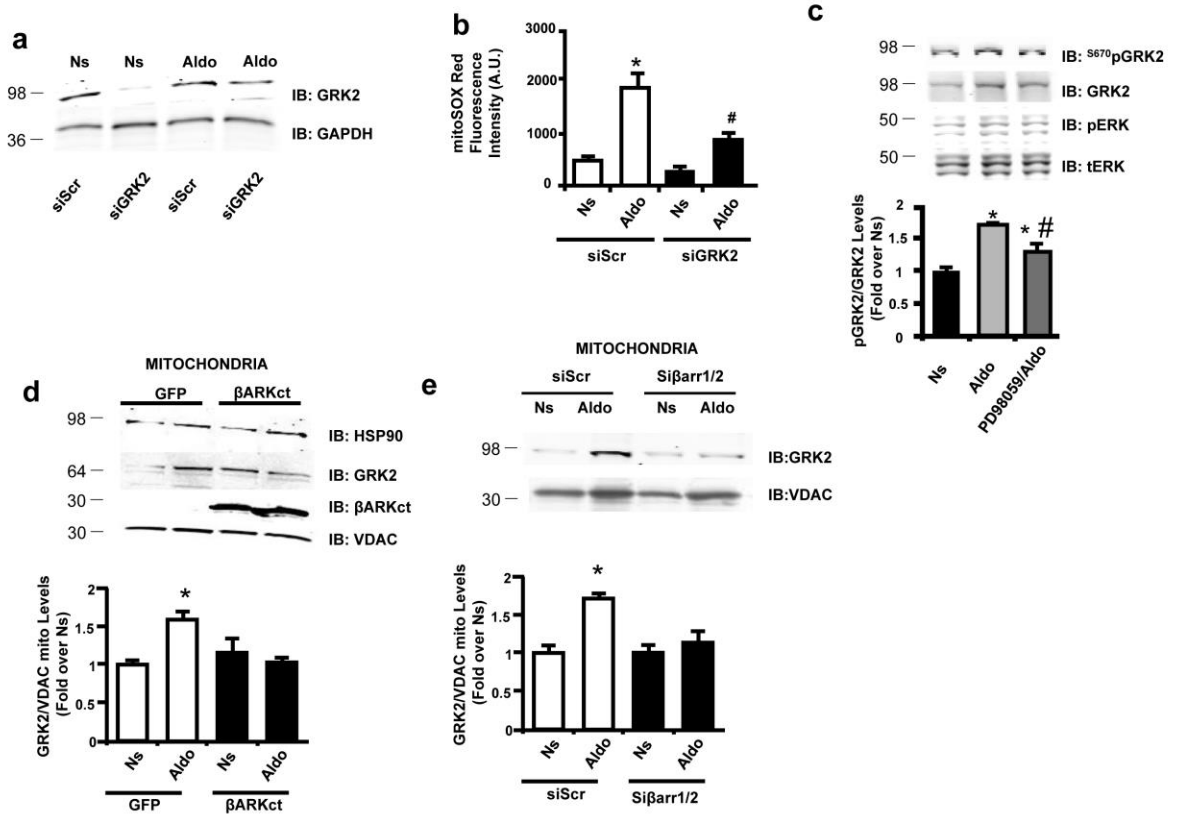

Supplementary Figure 2: GRK2/ERK/β-arrestins axis activation is absolutely required for aldosterone-dependent deleterious effects in NRVMs a) Representative immunoblot showing total GRK2 levels in NRVMs transfected with siRNA targeting GRK2 (siGRK2). Scrambled siRNA (siScr) were used as control. The cells were then Ns or stimulated for 24 hrs with Aldo (1 µM); **b)** Cumulative fluorescence data from (n=3) independent experiments of MitoSOX Red staining of NRVMs transfected with siGRK2 or siScr. The cells were then Ns or stimulated for 30 min with Aldo (1 µM); *,p<0.05 vs siScr Ns. **c)** Representative immunoblots (upper panels) and densitometric quantitative analysis (lower panel) of multiple (n=3) independent experiments to evaluate GRK2 phosphorylation (ser670) or ERK 1/2 phosphorylation levels in NRVMs Ns or stimulated with Aldo (1 µM) for 15 min. Prior to Aldo a group of cells was pre-treated with PD98059 (10 µM) for 30 min. Total ERK (tERK) and GRK2 are shown as loading controls; *,p<0.05 vs Ns. **d)** Representative immunoblot and densitometric analysis (lower) of multiple (n=3) independent experiment showing GRK2 and HSP90 levels in mitochondrial fractions purified from NRVMs infected with Ad-GFP (white bars) or Ad-βARKct (black bars). Myocytes were either Ns or stimulated with Aldo (1 µM) for 30 min. VDAC was used as a mitochondrial marker and loading control. *, p<0.05 vs GFP NS. **e)** Representative immunoblots (upper panels) and densitometric

quantitative analysis (lower panel) of multiple (n=3) independent experiments to evaluate GRK2 levels in mitochondrial fractions purified from NRVMs transfected with siRNAs targeting β-arrestin 1/2. siScr were used as control. NRVMs were either Ns or stimulated with Aldo (1 μM) for 30 min. VDAC was used as loading and mitochondrial purity control; *,$p<0.05$ vs siScr Ns. **b-c-d-e**) Statistical significance between groups was determined by one-way ANOVA with Bonferroni post-hoc correction. All data are shown as the means±s.e.m.

Supplementary Figure 3

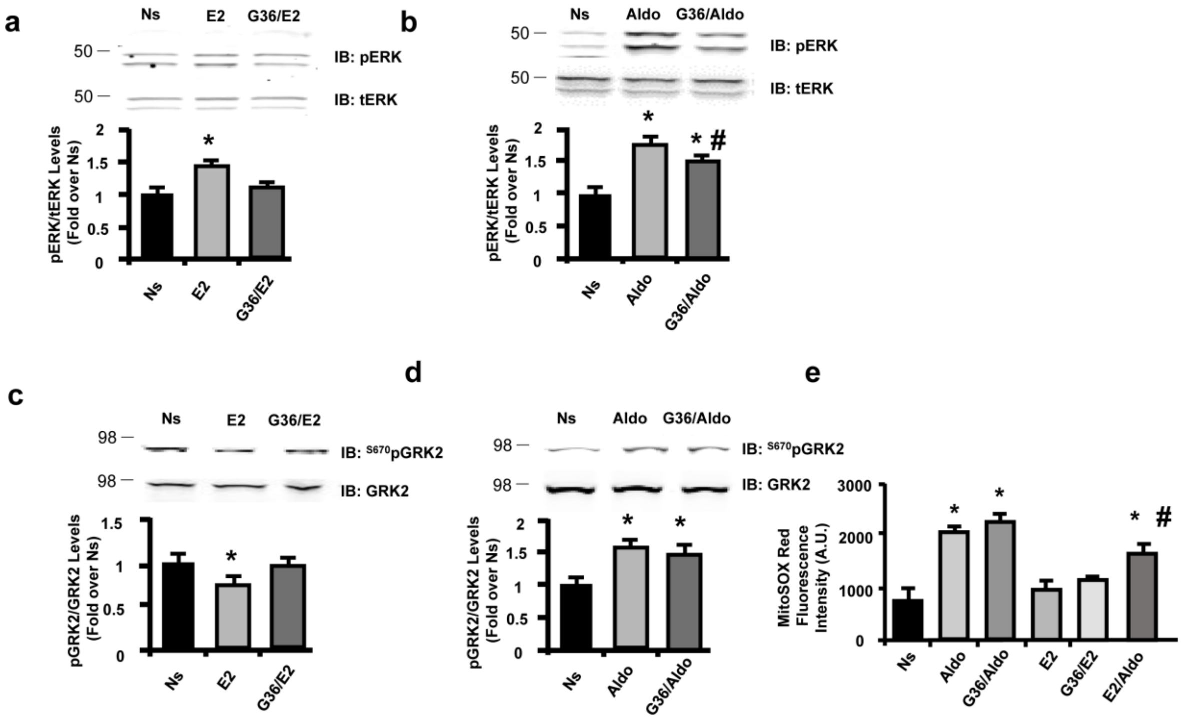

Supplementary Figure 3: Aldosterone activates a parallel GPER signaling that counteracts the negative GRK2 activity on ROS generation. **a-b)** Representative immunoblots (upper panels) and densitometric quantitative analysis (lower panel) of multiple (n=3) independent experiments to evaluate ERK 1/2 phosphorylation (pERK) as a ratio of activated ERK to total ERK (tERK) in NRVMs either unstimulated (Ns) or stimulated with 17β-estradiol (E2; 10 nM) or Aldo (1 µM) for 15 min. Also included are experiments where prior to E2 or Aldo treatment, myocytes were pre-treated with the GPER antagonist G36 (500 nM). *,$p<0.05$ vs Ns; #,$p<0.05$ vs Aldo. **c-d)** Representative immunoblots (upper panels) and densitometric quantitative analysis (lower panel) of multiple (n=3) independent experiments to evaluate GRK2 phosphorylation (ser670) in NRVMs that were Ns or stimulated with E2 (10 nM) or with Aldo (1 µM) for 15 min. Prior stimulation a group of cells was pre-treated with G36 (500 nM) for 30 min. Total GRK2 is shown as loading controls; *,$p<0.05$ vs Ns. **e)** Cumulative fluorescence data from (n=3) independent experiments of MitoSOX Red staining of NRVMs Ns or stimulated for 30 min with E2 (10 nM) or Aldo (1 µM); Prior stimulation a group of cells was pre-treated with G36 (500 nM) for 30 min. *,$p<0.05$ vs Ns; #,$p<0.05$ vs Aldo. **a-b-c-d-e**) Statistical significance between groups was determined by one-way ANOVA with Bonferroni post-hoc correction. All data are shown as the means±s.e.m.

Supplementary Figure 4

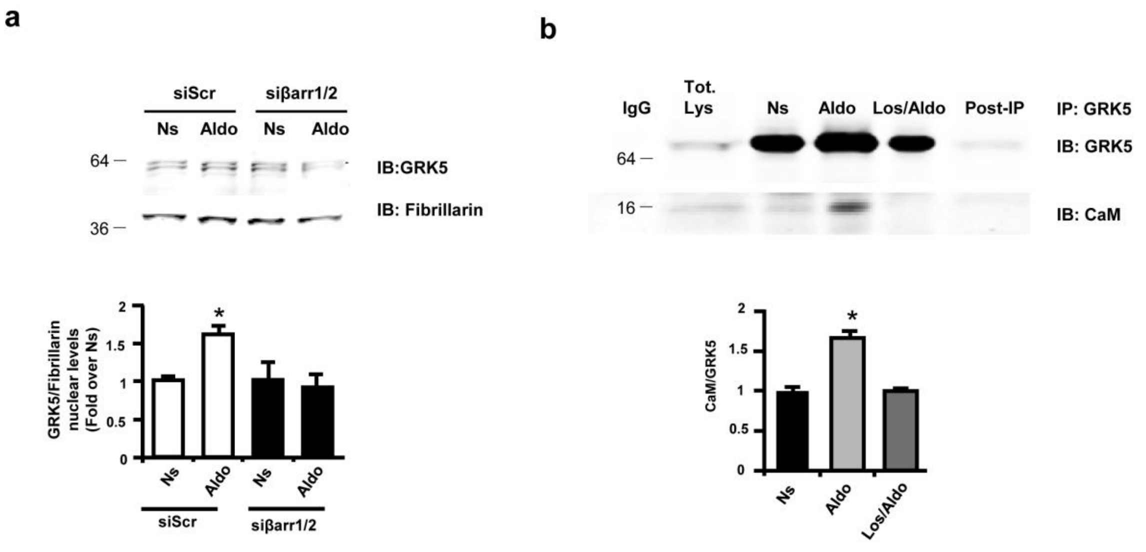

Supplementary Figure 4: β-arrestin recruitment and AT_1R activation after aldosterone stimulation are needed for GRK5 nuclear translocation a) Representative immunoblots (upper panels) and densitometric quantitative analysis (lower panel) of multiple (n=3) independent experiments to evaluate GRK5 levels in nuclear fractions purified from NRVMs transfected with siRNAs targeting β-arrestin 1/2. siScr were used as control. NRVMs were either Ns or stimulated with Aldo (1 µM) for 30 min. Fibrillarin was used as loading and mitochondrial purity control;*,p<0.05 vs siScr Ns. **b)** Representative panels (upper panels) and densitometric analysis (lower panel) of multiple (n=3) independent experiments of Co-IP assay in total lysates from NRVMs Ns or stimulated with Aldo (1 µM) for 30 minutes. Prior to stimulation a group of cells was pre-treated with Losartan (10 µM). Immunoprecipitated proteins (IP) for GRK5 were blotted with an antibody anti-Calmodulin (CaM) antibody; *,p<0.05 vs Ns. **a-b**) Statistical significance between groups was determined by one-way ANOVA with Bonferroni post-hoc correction. All data are shown as the means±s.e.m.

Supplementary Figure 5

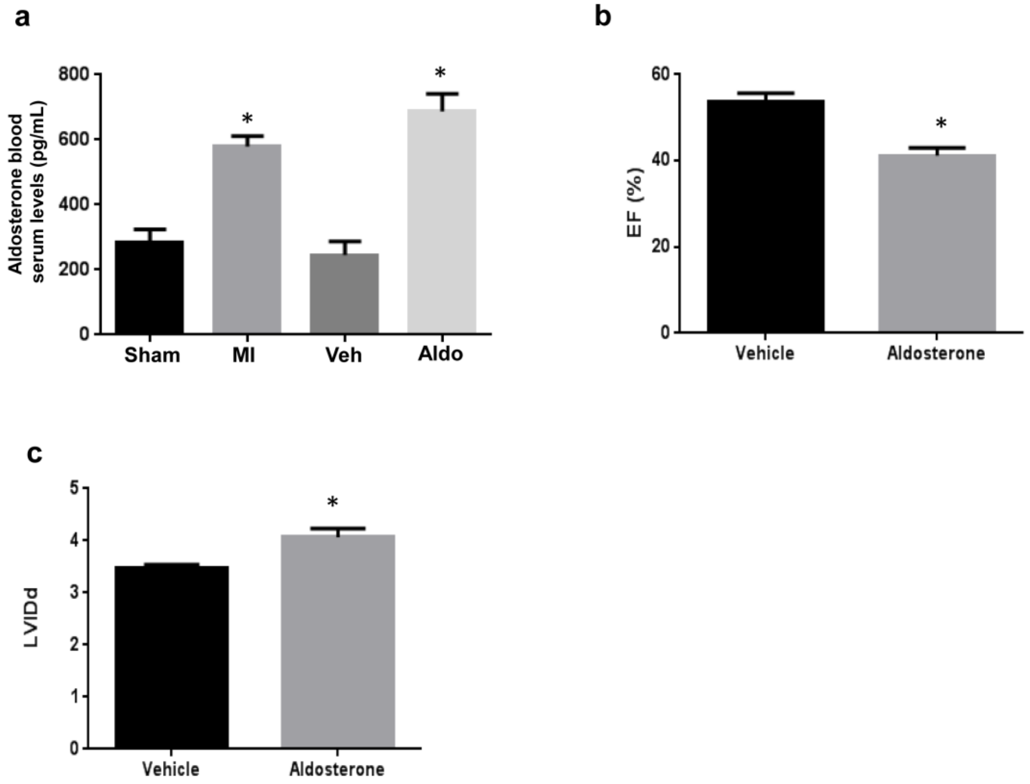

Supplementary Figure 5: a) Aldosterone blood serum levels measured by ELISA assay following either 4 weeks of mini-osmotic pump implantation (Aldo – 2ug/mouse/day) or 4 weeks after surgically induced myocardial infarction (MI) (n=5 each group). Sham operated mice or mice infused with saline (Vehicle) were used as controls. Statistical significance between groups was determined by one-way ANOVA with Bonferroni post-hoc correction*, $p<0.05$ vs Sham. **b-c)** Bar graphs showing the echocardiographic analysis of individual mice from αMHC-Cre mice after 4 weeks of Aldo (2 µg/day) or Veh (saline) treatment. Shown are measurements for (**b**) EF and (**c**) LVIDd. N=4 mice for each group. **b-c**) Statistical significance between groups was determined by Mann-Whitney exact test. *,$p<0.05$ vs Veh. All data are shown as the means±s.e.m.

Supplementary Figure 6

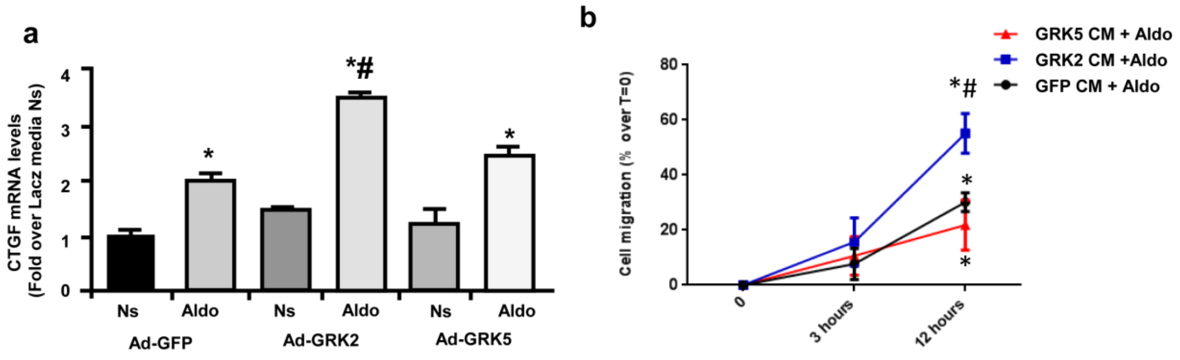

Supplementary Figure 6: GRK2 acting downstream aldosterone signaling in NRVMs is involved in secretion of CTGF and in the activation of cardiac fibroblasts. **a)** Bar graph showing quantitative data of qPCR experiments to evaluate CTGF mRNA levels in NRVMs infected with Ad encoding for GFP, GRK2 and GRK5. NRVMs were either Ns or stimulated with Aldo (1 µM) for 24 hours. *,$p<0.05$ vs GPF Ns. #,$p<0.05$ vs GFP Aldo; **b)** Cumulative data of multiple independent experiments (n=3) showing percentage of cell migration, evaluated by wound healing scratch assay. Confluent monolayers of neonatal rat cardiac fibroblasts were wounded at time 0 (T = 0). Cells were stimulate with conditioned media (CM) from NRMVs infected with Ad encoding for GFP, GRK2 and GRK5 and stimulated for 24 hours with Aldo (1 µM). The average rate of wound closure after 3 and 12 hours of wound healing was calculated; *,$p<0.05$ vs GFP T=0; #,$p<0.05$ vs GFP T=3h. **a-b**) Statistical significance between groups was determined by one-way ANOVA with Bonferroni post-hoc correction. All data are shown as the means±s.e.m.

Supplementary Figure 7

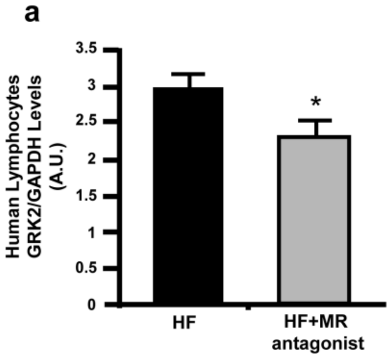

Supplementary Figure 7: Lymphocytes GRK2 protein levels are reduced human HF-patients after MR-antagonist treatment. Bar graphs showing cumulative data from immunoblots to evaluate GRK2 protein levels in human lymphocytes isolated from heart failure (HF) patients treated with (n=46) or without (n=81) MR antagonist Spironolactone (see Method section). GAPDH was used as loading control. *, $p<0.05$ vs HF. Statistical significance between groups was determined by the use of Student t test. GRK2 protein levels are expressed as mean ± standard deviation (SD).

Supplementary Figure 8

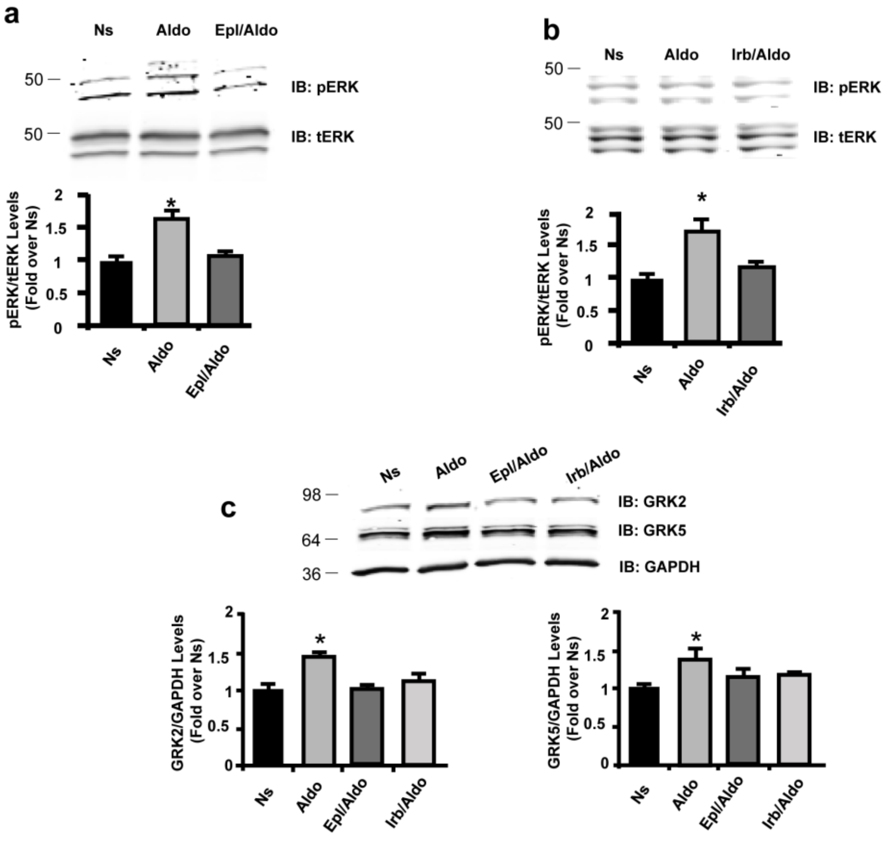

Supplementary Figure 8: Aldosterone-mediated activation of ERK and GRK2/5 is completely abolished by selective MR or AT_1R antagonism. a-b) Representative immunoblots (upper panels) and densitometric quantitative analysis (lower panel) of multiple (n=3) independent experiments to evaluate ERK 1/2 phosphorylation (pERK) as a ratio of activated ERK to total ERK (tERK) in NRVMs either unstimulated (Ns) or stimulated with Aldo (1 µM) for 15 min. Also included are experiments where prior Aldo treatment myocytes were pre-treated with selective MR antagonist eplerenone (Epl, 1 µM) or AT_1R antagonist irbesartan (Irb, 10 µM). *,$p<0.05$ vs Ns; **c)** Representative immunoblots (upper panels) and densitometric quantitative analysis (lower panel) of multiple (n=3) independent experiments to evaluate total GRK2 and GRK5 levels in NRVMs either unstimulated (Ns) or stimulated with Aldo (1 µM) for 15 min. Also included are experiments where prior Aldo treatment myocytes were pre-treated with selective MR antagonist Epl (1 µM) or AT_1R antagonist Irb (10 µM). GAPDH was used as loading control. *,$p<0.05$ vs Ns; **a-b-c**) Statistical significance between groups was determined by one-way ANOVA with Bonferroni post-hoc correction. All data are shown as the means±s.e.m.

Supplementary Figure 9

Uncropped blots used for the representative figures

Representative Figures 1a-b-c-d

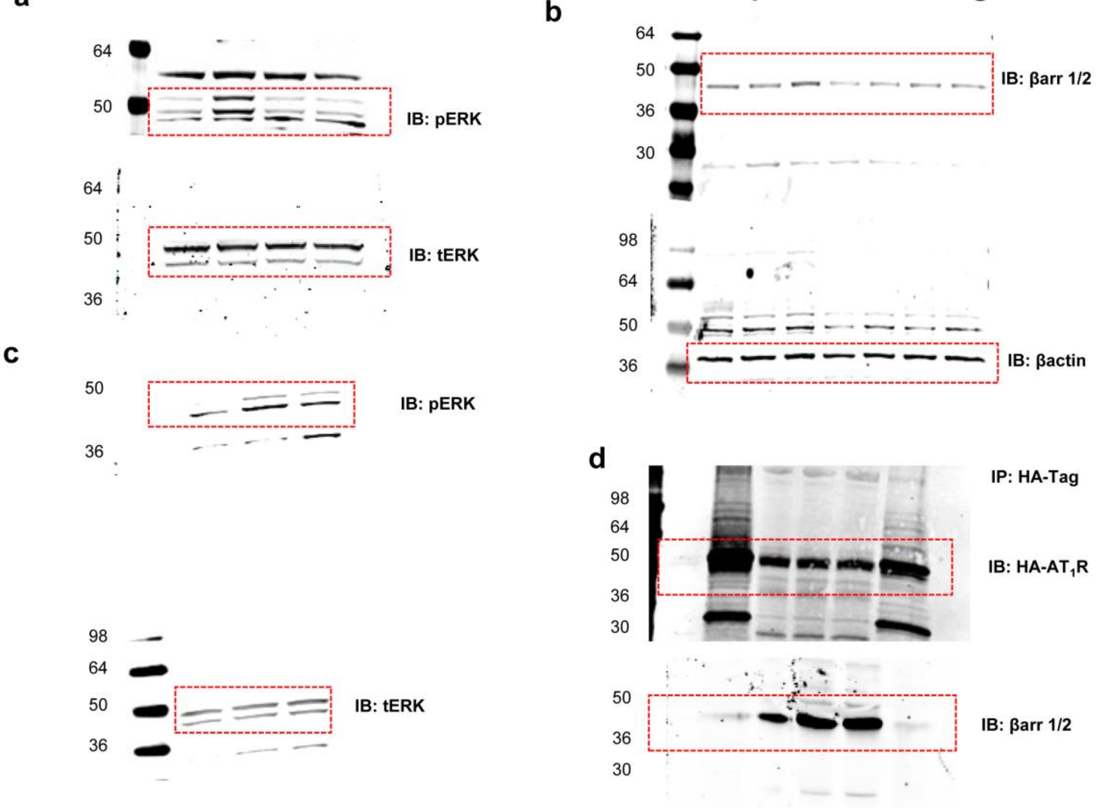

Representative Figures 2a-e

e

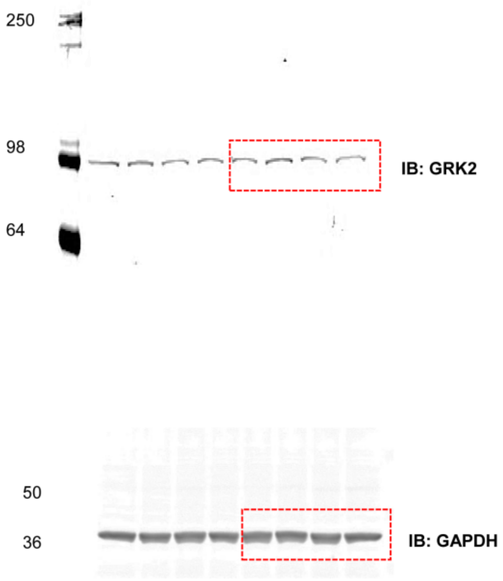

a

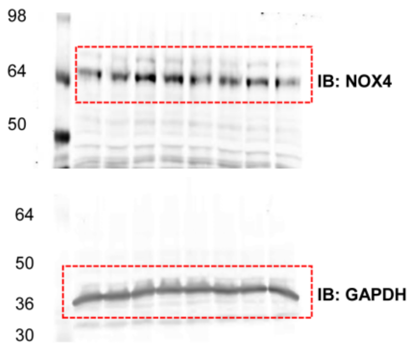

Representative Figures 3c-d-e

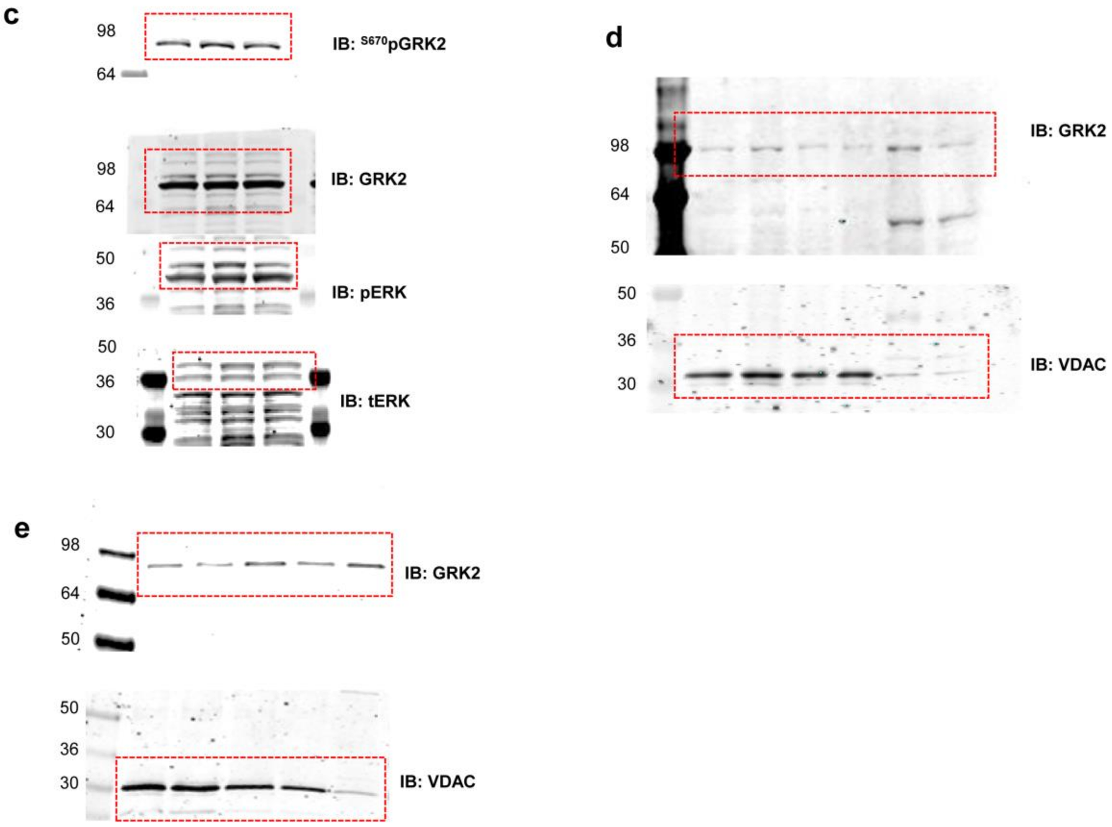

Representative Figures 4a-c

a

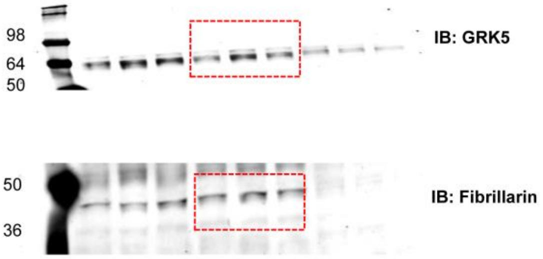

c

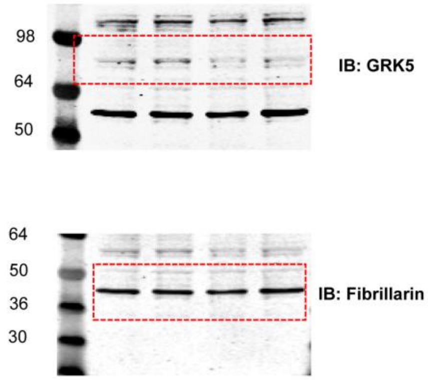

e

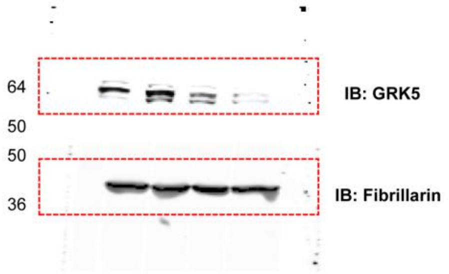

Representative Figures 5a-c

b

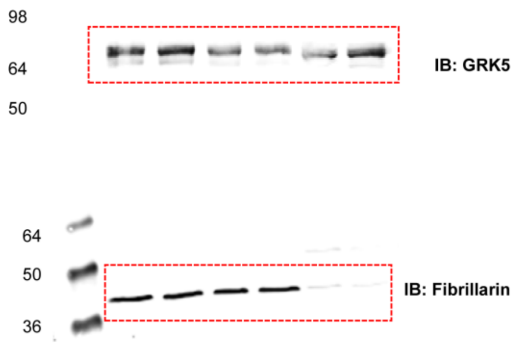

Representative Figures 6c-d-e-f

c

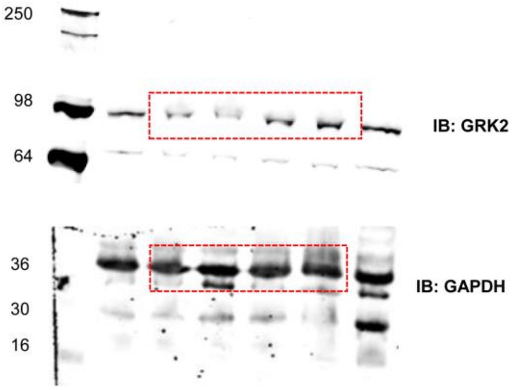

d

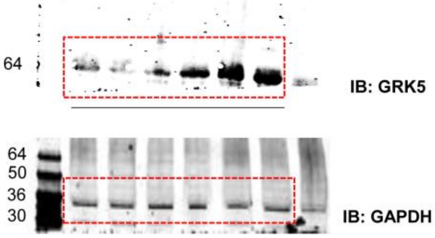

e

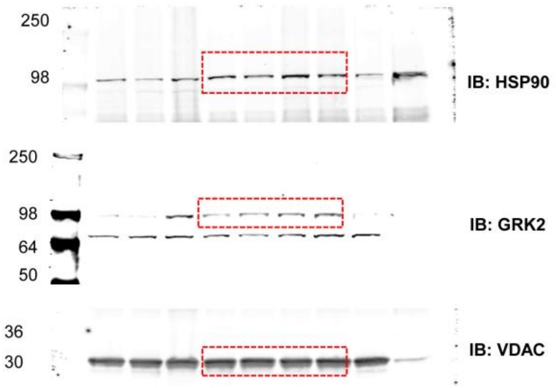

f

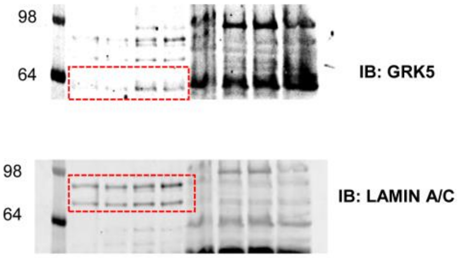

Representative Supplementary Figures 1a-b

a

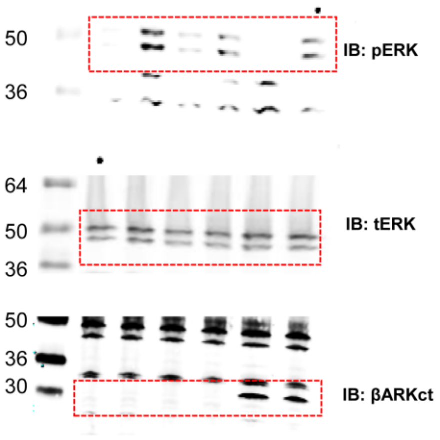

b

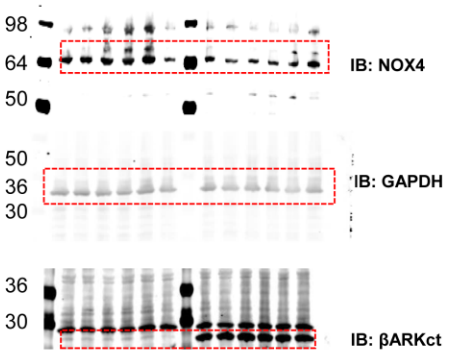

Representative Supplementary Figures 2a-c-d-e

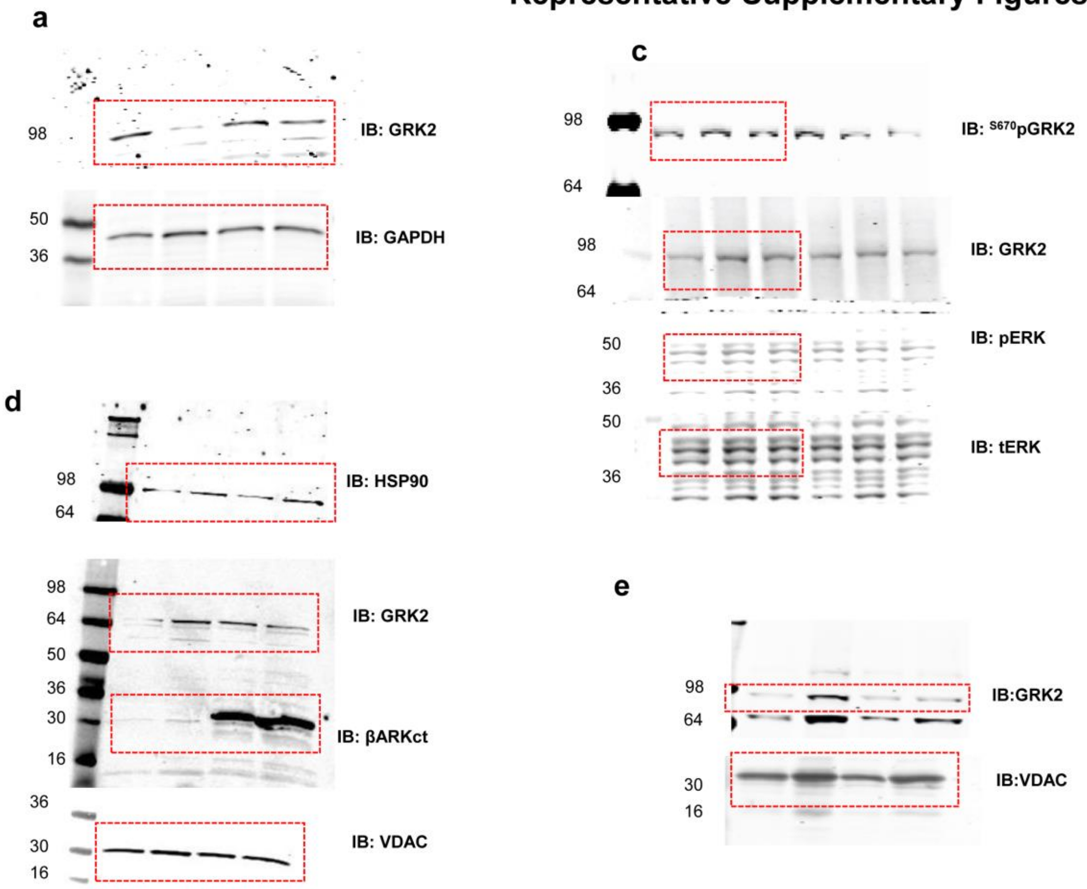

Representative Supplementary Figures 3a-b-c-d

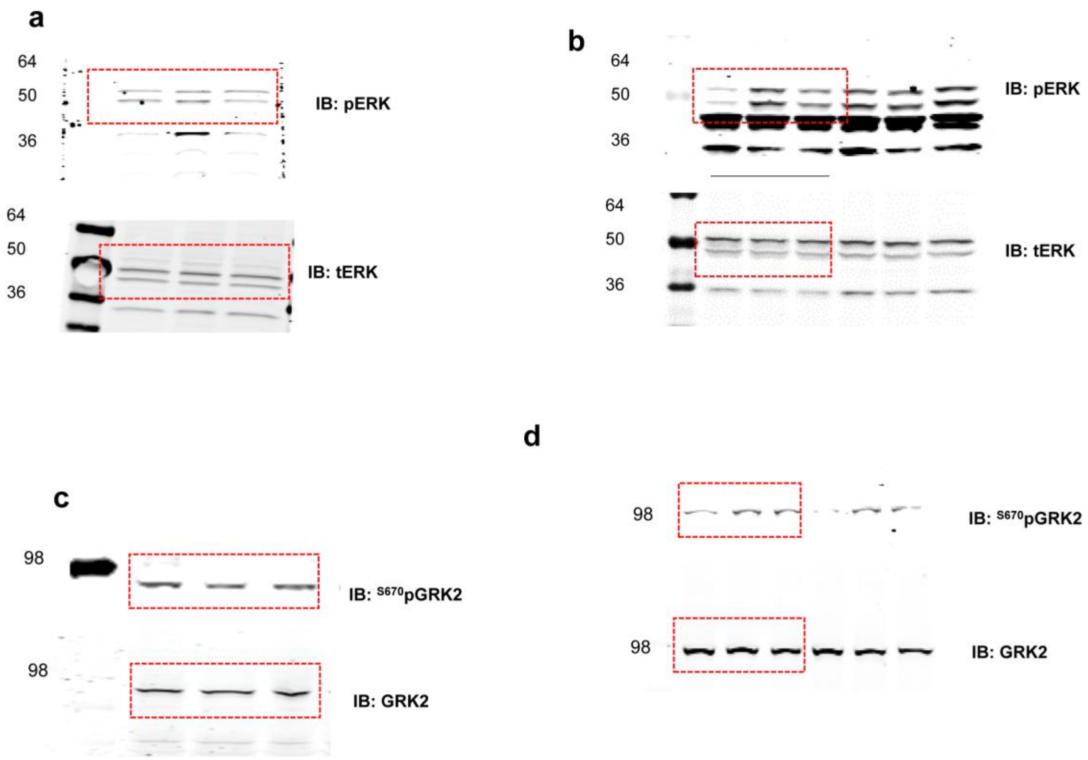

Representative Supplementary Figures 4a-b

a

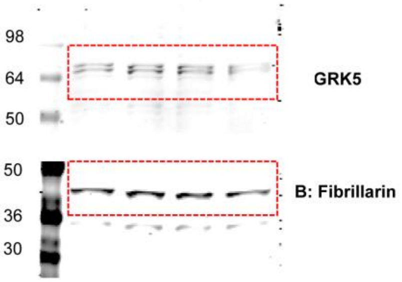

b

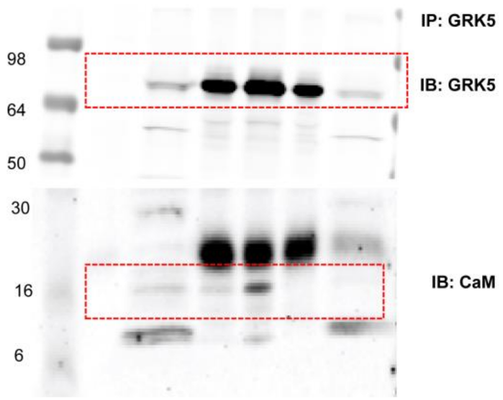

Representative Supplementary Figures 8a-b-c

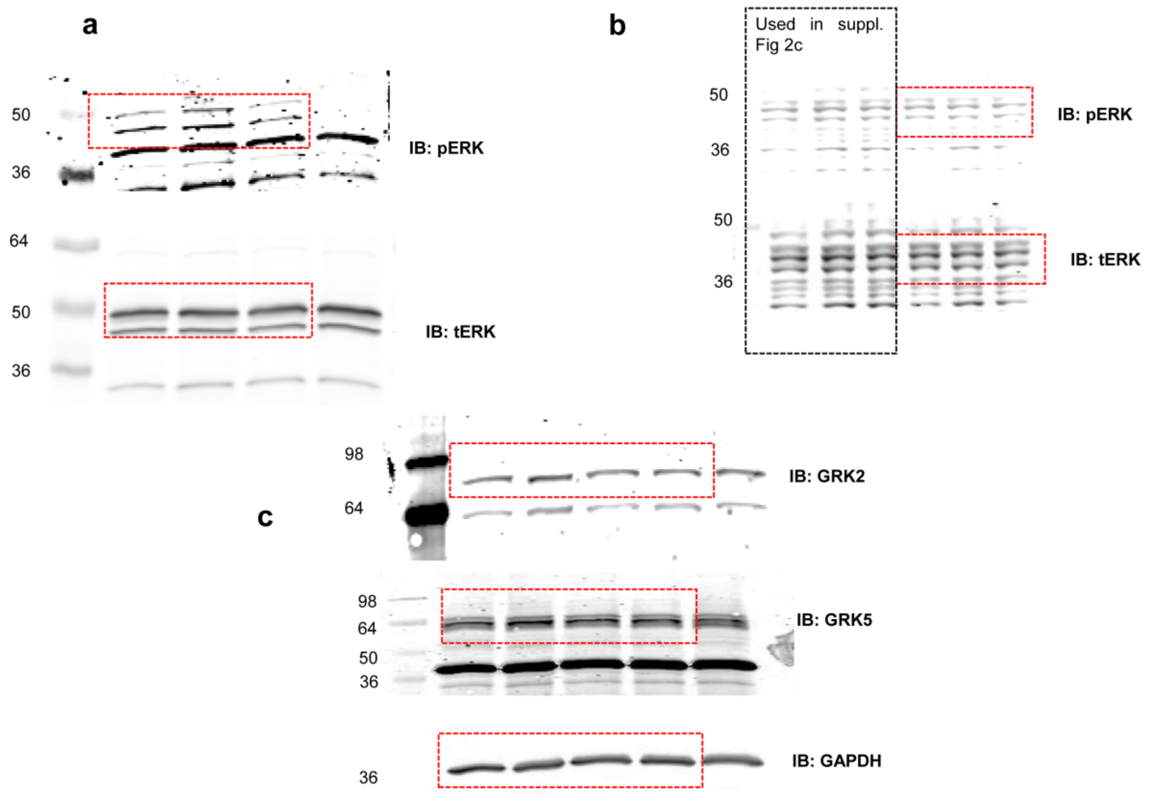

	HF<br>(n=81)	HF/Spironolactone<br>(n=46)	p value
Age	66.60 ± 10.37	64.24 ± 9.40	ns
Gender (Male, %)	68/81 (83.95 %)	35/46 (76.08 %)	ns
LVEF (%)	31.90 ± 8.16	30.80 ± 6.67	ns
NYHA class (%)	NYHA I=4 (4.93 %)<br>NYHA II=50 (61.72 %)<br>NYHA III=26 (32.09 %)<br>NYHA IV=1 (1.23 %)	NYHA I=1 (2.17 %)<br>NYHA II=24 (52.17 %)<br>NYHA III=21 (45.65)<br>NYHA IV=0 (0 %)	ns<br>ns<br>ns<br>ns
DM (%)	37/81 (45.67 %)	25/46 (54.34 %)	ns
Hypertension (%)	62/81 (76.54 %)	32/46 (69.56 %)	ns
ACE-I (%)	38/81 (46.91 %)	26/46 (56.52 %)	ns
ARBs (%)	27/81 (33.33 %)	12/46 (26.08 %)	ns
β-Blockers (%)	56/81 (69.13)	38/46 (82.60 %)	ns

Supplementary Table 1. Characteristics of HF-patients in the study population treated and non-treated with MR antagonist Spironolactone

DM, diabetes mellitus; LVEF, left ventricular ejection fraction; NYHA, New York Heart Association; ACE-I, angiotensin-converting-enzyme inhibitor; ARBs, angiotensin receptor blockers. Continuous variables are expressed as mean ± standard deviation (SD) and compared by the use of Student t test. Categorical variables are expressed as proportion and compared by use of $\chi 2$ test. p value is referred to difference between HF patients treated and non-treated with Spironolactone (see Methods section).
